# Habitual qat chewing and short-term response to conservative management of temporomandibular disorders: A prospective cohort study in conflict-affected Yemen

**DOI:** 10.1371/journal.pone.0362744

**Published:** 2026-11-13

**Authors:** Raji Mansoor Hajeb, Yousif Osman Yousif, Nader Ahmed Al-Aizari, Essam Ahmed Al-Moraissi

**Affiliations:** 1 Dentistry Department, Mahrah University, Mahrah, Yemen; 2 Faculty of Dentistry, University of Khartoum, Khartoum, Sudan; 3 Dentistry Department, Vision College in Riyadh, Riyadh, Saudi Arabia; 4 Faculty of Dentistry, Thamar University, Thamar, Yemen; UCLA School of Dentistry, UNITED STATES OF AMERICA

## Abstract

Qat (Catha edulis), chewed daily by a substantial proportion of Yemeni adults, contains cathinone, an amphetamine-like alkaloid that augments masticatory muscle activity. Observational evidence links the habit to temporomandibular disorders (TMD), yet none has examined how chewers respond once treated. A prospective cohort of 148 consecutive patients diagnosed by Research Diagnostic Criteria for Temporomandibular Disorders Axis I examination was followed at Sana’a University and Al-Kuwait Teaching Hospital between October 2018 and October 2020. Fifty-four (36.5%) were habitual qat chewers, defined as three or more sessions weekly. All received reassurance, education and self-care advice, to which the clinician added an NSAID, diazepam, amitriptyline or a stabilization splint as routine care. The outcome was a patient-reported global rating of change at three months, dichotomized as improved or not improved; every patient attended that visit. Improvement occurred in 47 of 54 chewers (87.0%) and 88 of 94 non-chewers (93.6%), a difference of 6.6 percentage points (95% CI −16.8 to 3.7; Fisher exact p = 0.23). The crude odds ratio for improvement among chewers was 0.47 (95% CI 0.15–1.42); it reached 0.92 (95% CI 0.26–3.29) once age and sex were entered, chewers being older and age the only consistent predictor of non-improvement. Treatment components did not differ between groups. Pain presented differently in chewers rather than simply more severely: 29.6% reported none and 24.1% severe pain, none falling in the mild band, so the bands differed in shape (p < 0.001) but not in rank (p = 0.22). Among those in pain, 38 of 40 chewers rated it moderate or severe against 52 of 82 non-chewers. Conservative management benefited nine patients in ten. Powered only for a difference of 16 percentage points or more, the study cannot exclude a clinically meaningful disadvantage to chewers; all adjusted estimates rest on 13 events and are hypothesis-generating.

## Introduction

Temporomandibular disorders (TMDs) constitute a heterogeneous group of conditions affecting the temporomandibular joint (TMJ), the masticatory muscles and the associated craniofacial structures [1]. They affect approximately 10–15% of adults worldwide, predominantly women in early to middle adulthood [2–4], and arise through some combination of biomechanical, psychological and neurobiological mechanisms [3,5]. First-line care is conservative, comprising patient education, self-care, behavioural modification and pharmacotherapy; although such management succeeds in the majority of patients, the response nevertheless varies with psychosocial, environmental and cultural circumstances [6–8].

Yemen presents an unusual configuration of those circumstances. The humanitarian crisis that began in 2015 has left high rates of anxiety, depression and post-traumatic stress disorder within the civilian population [9,10]. Psychological burden of this kind is, moreover, linked to the onset, persistence and exacerbation of TMD symptoms, plausibly through central sensitisation and increased masticatory muscle activity [11–14]. Superimposed upon this is a habit encountered in comparatively few other settings.

Qat chewing is practised daily by a large share of Yemeni adults [15,16]. The plant is transliterated both as qat and as khat; the terms are interchangeable, and qat is employed throughout the present paper except where a cited work adopts the alternative spelling. A bolus of leaves is retained in the buccal sulcus and chewed, generally on one side, for several hours during an afternoon session, and the habit carries a well-described burden of oral disease [17,18]. Its principal alkaloid, cathinone, is sympathomimetic and augments masticatory muscle activity [19,20]; furthermore, it interacts with several cytochrome P450 isoenzymes, thereby raising at least the theoretical possibility of altered metabolism of the drugs employed in TMD care [21,22]. Imaging corroborates a structural effect upon the joint, chronic chewers demonstrating osteoarthritic condylar change, joint effusion and disc displacement, most clearly after prolonged exposure [23,24].

What that literature does not address is treatment. A systematic review and meta-analysis published in 2026 pooled 13 observational studies and 3,526 participants and found chewers approximately two and a half times more likely to have TMD than non-chewers (odds ratio 2.48, 95% CI 1.20–5.09), while cautioning that the underlying evidence is heterogeneous and of low quality [25]. Every study within that review addressed whether the habit is associated with having TMD; none, however, examined what becomes of chewers once treated. Even the single prospective investigation, reported by Bakhadher et al. [26], followed 240 khat chewers in Aden by questionnaire and documented the prevalence of self-reported TMJ disorder at 34.2%, without a comparison group and without treatment. The present study was accordingly designed to address that gap by recruiting consecutive treatment-seeking patients with a clinical Axis I diagnosis, chewers and non-chewers alike, managing them conservatively and re-examining them at three months.

The question posed was whether habitual qat chewing is associated with a poorer short-term response to such management. Four baseline covariates were identified prior to analysis as the most probable confounders and were carried into the adjusted models: age, sex, self-reported emotional stress and self-reported tooth grinding.

## Materials and methods

### Study design and setting

This prospective observational cohort study followed consecutive patients receiving routine care within the Oral Medicine Department of Sana’a University and at Al-Kuwait Teaching Hospital, Sana’a, Yemen, between 1 October 2018 and 31 October 2020. Clinical management was not assigned by the investigators. The choice of drug, its dose and the decision to provide a splint rested with the treating oral medicine specialist and followed the department’s standing protocol for TMD; the investigators recorded treatment as delivered rather than determining it. As the study observed routine care rather than allocating it, prospective trial registration did not apply. Reporting follows the STROBE statement for cohort studies [27], and the completed checklist is provided as S1 Checklist.

### Ethics statement

The protocol was approved by the Medical Research Ethics Committee, Faculty of Dentistry, Sana’a University, Sana’a, Yemen (Approval No. 166, dated 15 September 2018) and by the Research Committee, Faculty of Dentistry, National Ribat University, Khartoum, Sudan. Approval was granted under the doctoral protocol title “Evaluation of the outcome of conservative management of temporomandibular disorders among patients attending Sana’a University and Al-Kuwait Teaching Hospital”, of which the present analysis forms a pre-planned component. All procedures conformed to the Declaration of Helsinki, and written informed consent was obtained from every participant prior to enrollment. Eighteen participants fell within the 10–20 year age band, and some of them were minors under Yemeni law. For every participant under 18 years, written consent was obtained from a parent or legal guardian together with the participant’s own assent, and the ethics committee approved the inclusion of minors.

### Participants

Consecutive patients aged 11–65 years presenting with at least one sign or symptom of TMD were screened. Enrolment required a diagnosis established by RDC/TMD Axis I examination together with the patient’s agreement to complete three months of conservative management. Patients were excluded where the presenting pain was judged to arise from a source other than the temporomandibular system, such as odontogenic pain, neuropathic pain, atypical facial pain, migraine or a primary headache disorder, and where tooth grinding was present without any accompanying TMD sign or symptom.

A total of 156 patients were screened between October 2018 and October 2020. Eight did not meet the eligibility criteria, and 148 were enrolled and diagnosed by RDC/TMD Axis I examination. Fifty-four were habitual qat chewers and 94 were not. All 148 attended the three-month visit; there was no loss to follow-up and no missing outcome data (Fig 1).

**Fig 1:**
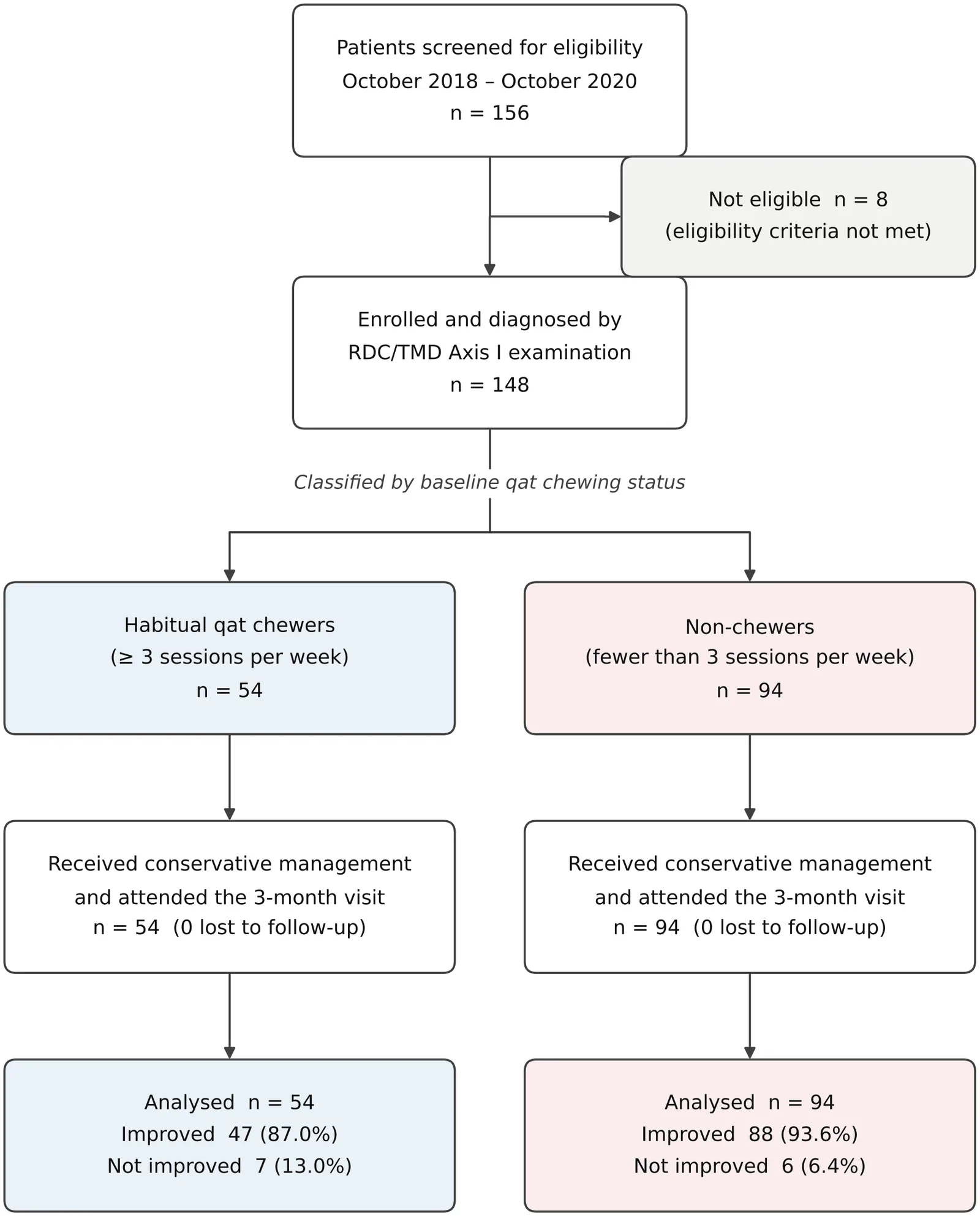
Participant flow through the study. Screening, enrolment, classification by baseline qat chewing status and follow-up of the whole cohort. Of 156 consecutive patients screened at the Oral Medicine Department of Sana’a University and Al-Kuwait Teaching Hospital between 1 October 2018 and 31 October 2020, 8 did not meet the eligibility criteria and 148 were enrolled following diagnosis by RDC/TMD Axis I examination. Fifty-four (36.5%) were classified as habitual qat chewers, defined as three or more chewing sessions per week, and 94 (63.5%) as non-chewers. All 148 participants attended the three-month follow-up visit; no participant was lost to follow-up in either group and no outcome data were missing. RDC/TMD, Research Diagnostic Criteria for Temporomandibular Disorders.

### Diagnostic criteria and clinical examination

Diagnoses followed the Research Diagnostic Criteria for Temporomandibular Disorders (RDC/TMD) Axis I [28] rather than the later Diagnostic Criteria (DC/TMD) [1]. The examination protocol, data collection forms and Arabic-language interview schedule of the parent doctoral project had been constructed and piloted against the RDC/TMD before recruitment opened, and examiner calibration was likewise conducted against that instrument; altering the instrument during a 25-month recruitment period would have rendered diagnoses non-comparable across the cohort. The DC/TMD had nonetheless been available for four years by 2018 and represents the current standard, a consideration taken up in the Limitations. Axis I findings were grouped as Group I (masticatory muscle disorders), Group II (disc displacements) and Group III (arthralgia, osteoarthritis and osteoarthrosis). Many patients satisfied criteria for more than one group, and the groups are accordingly reported non-exclusively.

A single oral medicine specialist experienced in TMD performed every examination, at baseline and at follow-up. Calibration against the RDC/TMD protocol preceded recruitment, and agreement upon repeat examination exceeded a Cohen’s kappa of 0.85 for all dichotomous items; TMJ pain on palpation, muscle tenderness, joint sounds, mandibular deviation and limitation of opening.

That same examiner recorded qat chewing status at baseline and the outcome at three months; assessment was consequently not blinded to exposure. In a setting where qat use is disclosed openly and its physical signs are readily visible, a single-examiner design could not realistically have achieved blinding. Any resulting bias would follow the examiner’s prior expectation, which within this department held that chewers fare worse, and would consequently tend to exaggerate rather than conceal a disadvantage among chewers.

Extraoral and intraoral inspection, digital palpation of the TMJ and of the cervical, extraoral and intraoral masticatory muscles, assessment of joint sounds during opening, closing, protrusion and excursion, and recording of mandibular deviation were conducted in the order specified by the RDC/TMD protocol. Mandibular range of motion was measured with a millimetre ruler between the maxillary and mandibular central incisal edges, with the vertical incisal overlap added; pain-free unassisted opening, maximum painful unassisted opening and maximum assisted opening were each recorded in millimetres.

### Exposure and covariates

Habitual qat chewing was ascertained at baseline by structured interview, patients reporting use in three or more sessions per week being classified as habitual chewers and all others as non-chewers. Duration of the habit was recorded in three bands (under 10 years, 10–20 years and over 20 years) and the habitual chewing side as left, right or bilateral. Session duration and the quantity chewed per session were not recorded; no participant, moreover, reported ceasing or reducing qat use during follow-up.

Pain intensity was rated by the patient at the baseline and final visits upon a written 11-point Numeric Rating Scale (0 = no pain) [29]. The integer score was transcribed into the study database only after collapsing it into the conventional bands of 0, 1–3 (mild), 4–6 (moderate) and 7–10 (severe); the underlying 0–10 value was retained solely on the paper clinical form. Mandibular opening was handled in the same way, the millimetre measurements being entered as the threshold categories used in the parent protocol (opening below or at least 40 mm, and pain-free opening below or at least 30 mm) rather than as continuous values. The electronic dataset therefore contains ordered categories alone, which is why no continuous baseline or follow-up measure can be analysed here; this constraint and its consequences are set out in the Limitations. Emotional stress and tooth grinding were recorded as present or absent by structured interview, grinding being taken as bruxism or clenching reported by the patient and corroborated by wear facets or morning masticatory fatigue. Sociodemographic variables; age band, sex, marital status, occupation, educational level and socioeconomic level, together with any history of head or neck trauma were recorded at the same visit.

### Management

Every patient received reassurance, an explanation of the condition and a self-care programme comprising a soft diet, avoidance of wide opening and of parafunctional loading, and moist heat or cold application at home. Drugs and splints were added to this regimen by the treating clinician according to the presenting complaint and the causative factors identified at examination. Three drug classes were employed: a non-steroidal anti-inflammatory drug, naproxen or diclofenac sodium, for inflammatory or arthrogenous pain; an anxiolytic, diazepam, where masticatory muscle hyperactivity or sleep disturbance predominated; and a tricyclic antidepressant, amitriptyline, for pain of long standing. Patients received either a single agent or a combination. A stabilization splint was provided in addition where parafunctional signs or persistent joint loading were present. The components delivered to each patient were entered in the study database and are reported by exposure group in Table 3.

Prescribed dose, dosing frequency and intended duration were written upon each patient’s treatment record rather than entered in the study database, and those records are not available in a form permitting analysis at cohort level. The appliance is likewise recorded only as a stabilization splint, without material, arch, occlusal coverage, wear instructions or review schedule. Safety data were similarly absent from the study variables. Patients were reviewed at three weeks, six weeks and three months, and an adverse effect reported at any visit would have been managed clinically and entered in the case record; no structured safety information, however, was extracted for analysis. No adverse-event rate can therefore be given, and none should be inferred from its absence. For a regimen incorporating a benzodiazepine and a tricyclic antidepressant this constitutes a substantive gap in the record, arising because treatment data were collected for clinical rather than research purposes.

Adherence to self-care advice, medication and splint wear was enquired about at each follow-up visit; the responses, however, were not captured as an analyzable variable and cannot be reported by group. The omission carries consequences beyond mere completeness, since differential adherence to the behavioural component constitutes the principal alternative explanation for any difference between chewers and non-chewers.

### Outcome

Patients were reviewed at three weeks, six weeks and three months. At the three-month visit the examiner recorded the patient’s global rating of change upon the four-point scale employed throughout the parent protocol; much better, better, improved or not improved, taking account of all three presenting complaints of pain, joint sounds and limitation of opening. For analysis the rating was dichotomised as improved, comprising the first three categories, versus not improved. A single global outcome of this kind constituted the only endpoint collected; separate subjective and objective outcomes were not recorded, and no composite requiring both was constructed. Since the rating originates with the patient and is entered by the examiner, it is described throughout as a patient-reported global rating rather than an objective measurement. Pain intensity upon the Numeric Rating Scale and maximum interincisal opening were re-recorded at the same visit but were not transcribed into the analysis dataset, precluding computation of a change score.

### Sample size

No a priori sample size calculation was performed. The sample comprises all eligible patients presenting over a 25-month recruitment window constrained by the security situation. With 54 chewers, 94 non-chewers and an improvement rate of 93.6% among non-chewers, the study possessed 80% power at a two-sided alpha of 0.05 to detect an absolute difference of 16.1 percentage points or larger, and power of 0.26 for the 6.6-point difference actually observed. It is adequately powered only for a large effect, a limitation bearing directly upon the interpretation of a null result.

### Statistical analysis

Analyses were performed in IBM SPSS Statistics version 22 (IBM Corp., Armonk, NY), with penalised-likelihood models fitted in Python 3.11 (statsmodels 0.14). Categorical variables are summarized as counts and percentages and compared using Fisher’s exact test, or the chi-square test where the table exceeded 2 × 2 and expected counts permitted; the chi-square test for trend was applied to the ordered age bands. The primary comparison is the unadjusted difference in improvement between chewers and non-chewers, expressed as an absolute risk difference with a 95% confidence interval, a risk ratio with a Katz logarithmic interval, and Fisher’s exact p-value. Two-sided p < 0.05 was taken as the threshold for statistical significance.

Thirteen patients failed to improve. A conventional five-predictor logistic regression would consequently carry 2.6 events per variable, well below the ten commonly recommended [30], and would yield unstable and biased estimates. All multivariable models were therefore fitted by Firth penalised likelihood [31], which reduces small-sample bias and produces finite estimates under near-separation, with profile penalized-likelihood confidence intervals and p-values derived from penalised likelihood-ratio tests. Three models are reported: unadjusted; a minimal model adjusting for age band and sex, at 4.3 events per variable; and the full five-predictor model adding emotional stress and tooth grinding, at 2.6 events per variable. All three rest upon 13 events and are accordingly hypothesis-generating rather than confirmatory. They are described as such wherever they appear, and no estimate drawn from them should be read as an effect size. Discrimination is summarized by the C-statistic and collinearity by variance inflation factors; sex-stratified estimates together with a Mantel-Haenszel odds ratio stratified by sex are furthermore reported as sensitivity analyses.

Baseline pain intensity was compared between groups in three complementary ways, since the banded variable carries ordinal information that a simple contingency test discards. The distribution across the four bands was compared by chi-square; the ordering of the bands was tested by Wilcoxon rank-sum; and the proportional-odds assumption was examined by estimating the odds ratio at each of the three cut-points. Duration of qat chewing was recorded only in bands and was not linked to the outcome within the analysis dataset; no analysis relating duration to treatment response was possible, and the band distribution is reported descriptively. Seven records (4.7%) proved internally discordant between the binary pain item and the Numeric Rating Scale band, three patients coded as having pain being assigned a band of 0 and four coded as pain-free being assigned a mild or moderate band. These records were left unaltered and both variables are reported as recorded; reanalysis excluding them left the conclusion unchanged (improvement in 45/52 chewers, 86.5%, versus 84/89 non-chewers, 94.4%; Fisher p = 0.13).

## Results

### Participants and baseline characteristics

Of 156 patients screened between October 2018 and October 2020, 148 were enrolled and all attended the three-month visit (Fig 1). Fifty-four (36.5%) were habitual qat chewers and 94 (63.5%) were not. One hundred and three participants (69.6%) were female and 45 (30.4%) male, and the majority were aged 21–30 years (91 patients, 61.5%).

The two groups differed sharply in age. No chewer fell within the youngest band, and 26 of 54 chewers (48.1%) were older than 30 compared with 13 of 94 non-chewers (13.8%) (p < 0.001). Chewers were moreover more often married (63.0% versus 21.3%, p < 0.001) and employed (37.0% versus 10.6%, p < 0.001), and less often educated to university level (38.9% versus 69.1%, p = 0.002), differences that track the age gradient. Sex, by contrast, was more evenly distributed: 20 of 54 chewers (37.0%) and 25 of 94 non-chewers (26.6%) were male, a gap of approximately ten percentage points (p = 0.20). Table 1 presents the full distribution.

**Table 1:** Baseline sociodemographic characteristics by qat chewing status.

Characteristic	Qat chewers (n = 54)	Non-chewers (n = 94)	p
**Age group, y**			<0.001
10–20	0 (0.0)	18 (19.1)	
21–30	28 (51.9)	63 (67.0)	
31–40	9 (16.7)	5 (5.3)	
41–50	8 (14.8)	6 (6.4)	
51–65	9 (16.7)	2 (2.1)	
**Sex**			0.198
Male	20 (37.0)	25 (26.6)	
Female	34 (63.0)	69 (73.4)	
Married	34 (63.0)	20 (21.3)	<0.001
Employed	20 (37.0)	10 (10.6)	<0.001
Low socioeconomic level	41 (75.9)	62 (66.0)	0.266
**Education**			0.002
No formal education	13 (24.1)	7 (7.4)	
Primary school	6 (11.1)	7 (7.4)	
Secondary school	14 (25.9)	15 (16.0)	
University	21 (38.9)	65 (69.1)	

Values are n (%) unless stated. p-values: Fisher’s exact test for 2 × 2 comparisons, chi-square test for age group and education. n = 148.

Presenting complaints were generally similar between the two groups (Table 2). Pain was reported by 122 patients overall (82.4%), joint sounds by 77 (52.0%) and limitation of opening below 40 mm by 45 (30.4%), none of which differed by exposure (p = 0.07, 0.86 and 0.85 respectively). Pain intensity, however, was distributed quite differently, and the nature of that difference deserves care.

**Table 2:** Baseline clinical characteristics and RDC/TMD Axis I diagnoses by qat chewing status.

Characteristic	Qat chewers (n = 54)	Non-chewers (n = 94)	p
Pain present	40 (74.1)	82 (87.2)	0.071
**Pain intensity (NRS band)**			<0.001
None (0)	16 (29.6)	9 (9.6)	
Mild (1–3)	0 (0.0)	32 (34.0)	
Moderate (4–6)	25 (46.3)	45 (47.9)	
Severe (7–10)	13 (24.1)	8 (8.5)	
Joint sounds	29 (53.7)	48 (51.1)	0.864
Limited mouth opening (<40 mm)	17 (31.5)	28 (29.8)	0.854
Headache	15 (27.8)	34 (36.2)	0.365
Emotional stress	43 (79.6)	86 (91.5)	0.045
History of head/neck trauma	11 (20.4)	8 (8.5)	0.045
Tooth grinding/clenching	9 (16.7)	36 (38.3)	0.006
Any parafunctional habit	13 (24.1)	45 (47.9)	0.005
**RDC/TMD Axis I group (non-exclusive)**			
Group I – masticatory muscle disorders	23 (42.6)	59 (62.8)	0.025
Group II – disc displacements	19 (35.2)	18 (19.1)	0.048
Group III – arthralgia/osteoarthritis	34 (63.0)	74 (78.7)	0.054

Values are n (%). Pain intensity was rated on an 11-point Numeric Rating Scale and entered into the dataset in the bands shown; p from chi-square test across the four bands. Across the ordered bands, Wilcoxon rank-sum p = 0.22. Axis I groups are non-exclusive: 82 patients met criteria for more than one group. n = 148.

Across the four bands the two distributions diverged sharply (chi-square p < 0.001), yet they did so in shape rather than in position. Chewers were over-represented at both extremes. Sixteen of 54 (29.6%) reported no pain at all against 9 of 94 non-chewers (9.6%), while 13 (24.1%) reported severe pain against 8 (8.5%). Not one chewer fell in the mild band, which held 32 of 94 non-chewers (34.0%). The ordering of the bands consequently carries no overall shift. The median band was moderate in both groups, a Wilcoxon rank-sum test across the ordered bands gave p = 0.22, and the odds ratio reversed direction across the three cut-points, from 0.25 for any pain versus none, through 1.84 for moderate-or-severe versus mild-or-none, to 3.41 for severe versus the rest. The proportional-odds assumption therefore fails, and no single ordinal summary would be honest. Restricted to the 122 patients who reported any pain at all, the contrast is unambiguous: 38 of 40 chewers (95.0%) rated that pain moderate or severe, against 52 of 82 non-chewers (63.4%) (p < 0.001).

Several risk factors were distributed unexpectedly. Emotional stress was near-universal yet slightly less often reported by chewers (79.6% versus 91.5%, p = 0.045), as were tooth grinding (16.7% versus 38.3%, p = 0.006) and any parafunctional habit (24.1% versus 47.9%, p = 0.005). A history of head or neck trauma, conversely, ran in the opposite direction (20.4% versus 8.5%, p = 0.045). Axis I diagnoses likewise differed by group, disc displacements proving more frequent among chewers (35.2% versus 19.1%, p = 0.048), masticatory muscle disorders less frequent (42.6% versus 62.8%, p = 0.025), and arthralgia and osteoarthritis similarly distributed (63.0% versus 78.7%, p = 0.054).

### Treatment received

Treatment was distributed almost identically across the two groups (Table 3). All 148 patients received reassurance, education and self-care advice. Pharmacotherapy of any kind was given to 42 chewers (77.8%) and 80 non-chewers (85.1%) (p = 0.27). Within that total, an NSAID was prescribed to 74.1% and 83.0% (p = 0.21), an anxiolytic to 53.7% and 61.7% (p = 0.39) and amitriptyline to 20.4% and 22.3% (p = 0.84). A stabilization splint was fitted to 13 chewers (24.1%) and 24 non-chewers (25.5%) (p = 1.00). No component differed by exposure group, so neither the overall intensity of treatment nor the choice among its sub-modalities can account for the outcome comparison that follows. Adherence to that treatment is a separate question, and one the dataset cannot answer; it is taken up in the Limitations.

**Table 3:** Components of conservative management received, by qat chewing status.

Treatment component	Qat chewers (n = 54)	Non-chewers (n = 94)	p
Reassurance, education and self-care	54 (100.0)	94 (100.0)	1.000
Any pharmacotherapy	42 (77.8)	80 (85.1)	0.270
NSAID (naproxen or diclofenac sodium)	40 (74.1)	78 (83.0)	0.209
Anxiolytic (diazepam)	29 (53.7)	58 (61.7)	0.388
Tricyclic antidepressant (amitriptyline)	11 (20.4)	21 (22.3)	0.838
Stabilisation splint	13 (24.1)	24 (25.5)	1.000

Values are n (%); p from Fisher’s exact test. Components are not mutually exclusive. Treatment was assigned by the treating clinician on clinical grounds, not by the investigators.

### Short-term response

At three months, 135 of 148 patients (91.2%) reported improvement: 47 of 54 chewers (87.0%) and 88 of 94 non-chewers (93.6%). The absolute difference of 6.6 percentage points in favour of non-chewers carried a 95% confidence interval from −16.8 to 3.7, the risk ratio for improvement was 0.93 (95% CI 0.83–1.04) and Fisher’s exact test yielded p = 0.23. The crude odds ratio for improvement among chewers was 0.47 (95% CI 0.15–1.42, p = 0.09). These data are compatible with no difference between the groups; the interval is wide, however, and a disadvantage to chewers of as much as 17 percentage points is equally compatible with them (Table 4, Fig 2).

**Table 4:** Short-term response to conservative management by qat chewing status.

Outcome	Qat chewers (n = 54)	Non-chewers (n = 94)	Effect estimate (95% CI)	p
Improved at 3 months	47 (87.0)	88 (93.6)	0.93 (0.83–1.04)	0.23
Not improved	7 (13.0)	6 (6.4)	—	—
Absolute difference (percentage points)			−6.6 (−16.8 to 3.7)	
Odds ratio for improvement (Firth penalised)			0.47 (0.15–1.42)	0.09

Outcome is the patient-reported global rating of change at three months, dichotomised as improved versus not improved. RR = risk ratio for improvement (Katz logarithmic 95% CI); OR = odds ratio for improvement; p from Fisher’s exact test.

**Fig 2:**
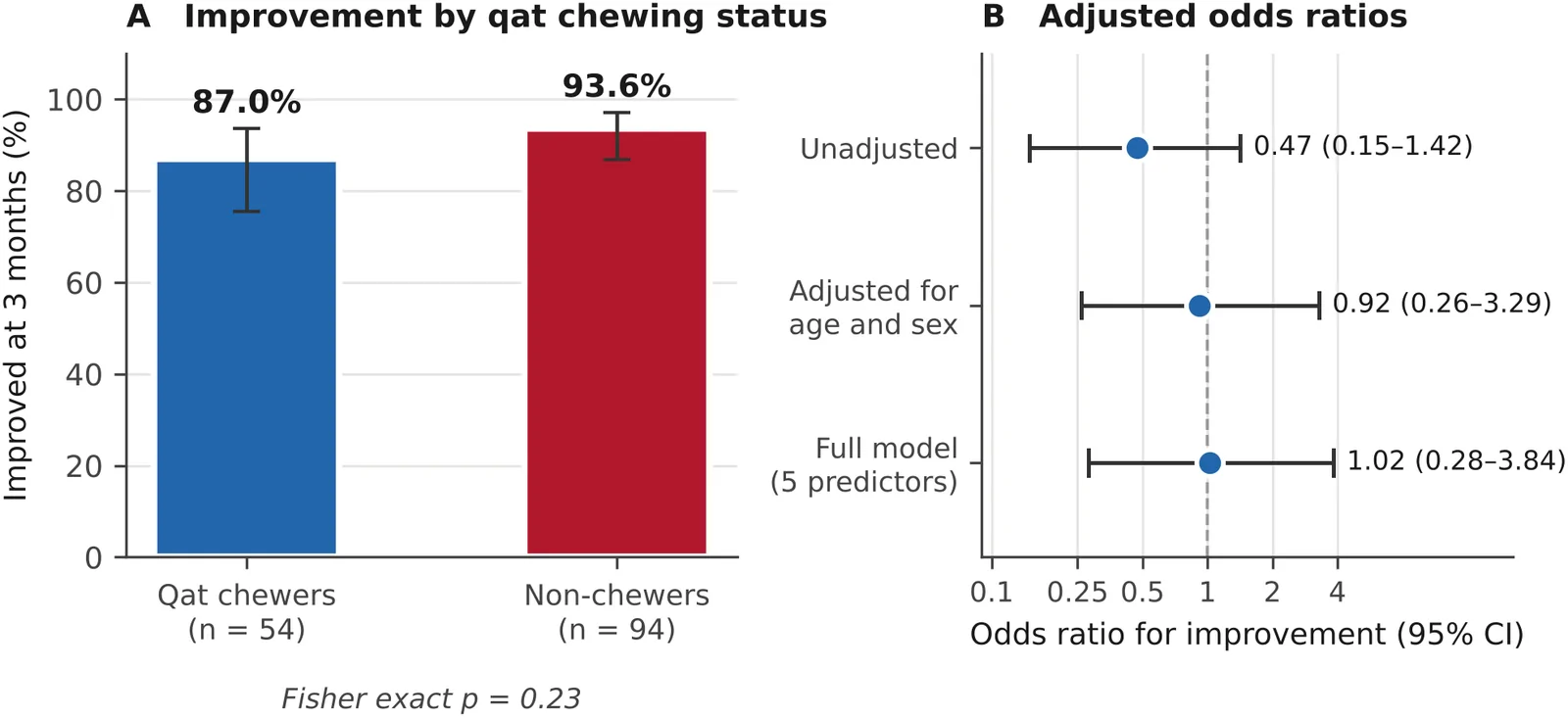
Short-term response to conservative management by qat chewing status. (A) Proportion of patients recorded as improved at the three-month visit, shown separately for habitual qat chewers and non-chewers, with 95% Wilson confidence intervals. Improvement occurred in 47 of 54 chewers (87.0%) and in 88 of 94 non-chewers (93.6%); Fisher’s exact p = 0.23. (B) Odds ratio for improvement among qat chewers relative to non-chewers, estimated by Firth penalised logistic regression and shown unadjusted, adjusted for age band and sex, and from the full five-predictor model additionally including emotional stress and tooth grinding. Points are point estimates and horizontal bars are 95% profile penalised-likelihood confidence intervals; the vertical dashed line marks the null value of 1.0. All three estimates rest upon 13 non-improvement events and are hypothesis-generating. CI, confidence interval; OR, odds ratio.

Neither sex stratum demonstrated a signal. Among men, 17 of 20 chewers (85.0%) improved against 22 of 25 non-chewers (88.0%) (p = 1.00); among women, 30 of 34 (88.2%) against 66 of 69 (95.7%) (p = 0.22). The Mantel-Haenszel odds ratio stratified by sex was 0.50, close to the crude estimate, as would be anticipated where sex is only weakly associated with the exposure. Variance inflation factors within the full model ranged from 1.05 to 1.31, indicating that collinearity between qat chewing and sex was not a feature of these data.

### Multivariable analysis (hypothesis-generating)

The models that follow rest upon 13 non-improvement events and are hypothesis-generating; they are presented to show the direction in which adjustment moves the crude estimate, not to quantify an effect. Firth penalised models are presented in Table 5. Unadjusted, qat chewing carried an odds ratio for improvement of 0.47 (95% CI 0.15–1.42, p = 0.09). The addition of age band and sex removed the association altogether, moving the estimate to 0.92 (95% CI 0.26–3.29, p = 0.34); the full five-predictor model yielded 1.02 (95% CI 0.28–3.84, p = 0.36), with neither emotional stress (1.15, 95% CI 0.19–4.96) nor tooth grinding (1.40, 95% CI 0.36–6.78) associated with the outcome.

**Table 5:** Firth penalised logistic regression for improvement at three months (hypothesis-generating).

Predictor	Odds ratio for improvement	95% CI	p
**Model 0 — unadjusted (EPV 13.0)**			
Qat chewing (yes vs no)	0.47	0.15–1.42	0.09
C-statistic	0.60		
**Model 1 — minimal, pre-specified (EPV 4.3)**			
Qat chewing (yes vs no)	0.92	0.26–3.29	0.34
Male sex	0.42	0.13–1.37	0.08
Age (per one band increase)	0.52	0.31–0.85	0.002
C-statistic	0.73		
**Model 2 — full model as originally specified (EPV 2.6)**			
Qat chewing (yes vs no)	1.02	0.28–3.84	0.36
Male sex	0.40	0.11–1.34	0.08
Age (per one band increase)	0.52	0.31–0.85	0.002
Emotional stress (yes vs no)	1.15	0.19–4.96	0.53
Tooth grinding (yes vs no)	1.40	0.36–6.78	0.33
C-statistic	0.77		

Outcome: improvement at three months. Estimates from Firth penalised likelihood; 95% CIs are profile penalized-likelihood intervals; p from penalised likelihood-ratio tests. EPV = events per variable (13 non-improvement events). Age modelled as ordered band (1 = 10–20 y through 5 = 51–65 y). All models are hypothesis-generating and none should be read as an effect size.

Age, notably, was what predicted the outcome. Each one-band increase carried an odds ratio for improvement of 0.52 (95% CI 0.31–0.85, p = 0.002), and the gradient across bands proved monotonic: 94.4% of patients aged 10–20 years improved, 95.6% of those aged 21–30, 85.7% aged 31–40, 78.6% aged 41–50 and 72.7% aged 51–65 (trend p = 0.003). Since chewers within this cohort were markedly the older group, age accounts for the crude association. The C-statistic rose from 0.60 for the exposure alone to 0.73 with age and sex and 0.77 for the full model; at 13 events, however, these values are unstable and are not interpreted further.

Among the 54 chewers, 17 (31.5%) reported chewing for under 10 years, 23 (42.6%) for 10–20 years and 14 (25.9%) for more than 20 years. Response by duration could not be examined, for the reason stated in the Methods.

## Discussion

Conservative management performed well within this cohort, 91.2% of patients reporting improvement at three months, and no evidence emerged that habitual qat chewing reduced that response. That statement needs its precise sense. Chewers improved 6.6 percentage points less often than non-chewers, and the confidence interval around that difference runs from a 16.8-point disadvantage to a 3.7-point advantage; the study is therefore unable to exclude a clinically meaningful disadvantage to chewers, and the appropriate reading is an absence of evidence rather than evidence of absence. The crude odds ratio of 0.47 appeared suggestive until age entered the model, whereupon it disappeared, chewers being considerably older and age the one variable that predicted non-improvement with any consistency. Since that observation comes from a model resting on 13 events, it is hypothesis-generating and nothing more.

The baseline pain finding is more interesting than a simple statement of greater severity would suggest, and it should not be summarized that way. Chewers occupied both ends of the scale: almost a third reported no pain at all, roughly a quarter reported severe pain, and not one fell in the mild band. Across the ordered bands there was consequently no shift in location at all (rank-sum p = 0.22), and the odds ratio reversed direction from one cut-point to the next. What differs between the groups is the shape of the distribution rather than its centre. Among patients who did present in pain, however, that pain was moderate or severe in 95.0% of chewers against 63.4% of non-chewers, and it is to this conditional contrast, not to any overall elevation of severity, that the subsequent discussion refers.

The 2026 meta-analysis by Al-Qadhi and colleagues pooled 13 observational studies and reported that chewers were roughly two and a half times as likely to have TMD (OR 2.48, 95% CI 1.20–5.09), although the pooled evidence was graded low in quality [25]. The conditional severity finding reported here is broadly consistent with that literature, although the bimodal distribution observed in the present cohort has no counterpart in it. The outcome finding answers a question none of those studies posed. Bakhadher et al. [26] prospectively followed 240 chewers in Aden but documented only the prevalence of self-reported TMJ disorder, without a comparison group and without treatment. Almashraqi et al. demonstrated osteoarthritic condylar change on CBCT, together with effusion and disc displacement on MRI, in chronic chewers [23,24]; disc displacement was likewise more frequent among chewers in the present cohort (35.2% versus 19.1%). Those structural findings describe what years of the habit may do to a joint; they say little about how that joint responds to a short course of an NSAID and three months of self-care. The present data suggest that the two questions carry different answers.

The mechanistic account customarily offered for qat and TMD is plausible yet remains untested here. Chewing is prolonged, largely unilateral and involves sustained clenching, which alters loading and neuromuscular activation [32], and cathinone is sympathomimetic, raising muscle activity and pain perception [15,33]. The same alkaloid inhibits or induces several CYP isoenzymes, among them CYP1A2, CYP2A6, CYP2D6, CYP3A4 and CYP3A5, and could in principle alter the metabolism of NSAIDs, diazepam and amitriptyline [21,22,34,35]. None of these variables was measured within the present study. No cathinone assay was performed, muscle activity was not quantified, drug levels were not obtained, and response was not examined by drug class. The pharmacokinetic interaction thus remains a hypothesis this setting raises rather than one these findings support; indeed, the data are mildly unhelpful to it, since a materially reduced drug efficacy would have left chewers receiving pharmacotherapy worse off, whereas near-identical treatment produced near-identical results.

A more parsimonious reading, and one that remains conjectural, holds that qat chewing is associated with how TMD presents rather than with resistance to its treatment. Such an interpretation would accommodate both the imaging literature and the meta-analytic association, and it carries a different clinical prediction: among chewers who present in pain, that pain should be expected to be more intense, rather than their response to treatment being poorer. The present study was not designed to test that prediction, and nothing here establishes it.

Two alternatives merit attention. The first is differential adherence to the behavioural component of care. Every patient was advised to adopt a soft diet and avoid parafunctional loading, and chewing qat for several hours daily itself constitutes a departure from that advice. Had chewers fared worse, behaviour would have explained the finding at least as well as pharmacology, and the two could not have been separated, since adherence was never captured as an analyzable variable. The outcome was null, which weakens this alternative alongside the primary hypothesis; the inability to quantify adherence nevertheless remains a genuine gap. The second alternative, confounding by treatment, can be addressed with data: splint provision, NSAID use, anxiolytic use and amitriptyline use proved statistically indistinguishable between groups, so the association reported here is not an artefact of chewers having been treated differently.

A third consideration concerns the examiner rather than the patients. One clinician recorded qat chewing status at baseline and the global rating of change at three months, and was necessarily aware of the former when assigning the latter. Calibration was strong for the physical signs (kappa above 0.85), but calibration cannot correct a bias of expectation, and the outcome in question was a judgement rather than a measurement. The departmental expectation was that chewers fare worse, so any such bias would have widened the apparent gap between the groups rather than narrowed it. That the observed gap was nonetheless compatible with chance makes the null finding somewhat harder to attribute to this source; had the result been positive, it would have been difficult to interpret at all. The absence of blinding remains a genuine weakness of the design, and a blinded assessor is the single change that would most improve a repeat of this study.

Emotional stress was reported by 87.2% of this cohort, high even for a TMD clinic and consistent with a population living through a war [9,36]. Prolonged displacement, economic collapse and insecurity are associated with anxiety and depressive symptoms in Yemen [10], and psychological distress is well established as a prognostic factor in TMD, both as a correlate of Axis I diagnosis and as a predictor of outcome [37–42]. Stress did not predict non-improvement in these data; that finding, however, carries little weight. A variable present in 87% of a cohort possesses almost no discriminatory power, and this one was captured as a yes-or-no answer to a structured question rather than by a validated instrument, RDC/TMD Axis II having not been administered. No inference regarding the role of stress can rest upon such a measure, and none is drawn here.

Nutrition may constitute a further pathway, albeit one the present study cannot examine. Long-term qat use has been associated with undernutrition, a meta-analysis of Ethiopian data placing the pooled odds ratio at 1.53 (95% CI 1.09–2.16) [43], and within a population already facing food insecurity such deficits could plausibly affect tissue recovery. No nutritional variable was measured.

Recruitment ran to 31 October 2020 and consequently overlapped the first year of the COVID-19 pandemic, which together with the security situation limited the number of patients who could be reached and contributed to the modest sample size. Follow-up completeness was nevertheless unaffected: an outcome was recorded at three months for all 148 enrolled patients, with no missing data [AUTHOR TO CONFIRM: whether recruitment was formally suspended at any point during 2020, and for how long, so that the interruption can be described precisely].

### Strengths and limitations

The study possesses several strengths. It was prospective and applied to consecutive treatment-seeking patients. Follow-up at three months was complete, with no attrition in either group; one calibrated examiner performed every assessment; and the analysis includes the full enrolled cohort, achieved within a setting where research of any kind is difficult to conduct.

Four limitations bear directly upon the interpretation of the null result and are set out first.

Power is the most obvious. The study could detect a difference of 16 percentage points or more with 80% certainty, whereas the difference observed was 6.6 points. A clinically meaningful difference of ten points would have been missed more often than not, so every statement that qat chewing was not associated with a poorer response means only that this cohort was unable to exclude such a difference.

The measurement of severity is the most consequential. Pain intensity was rated on an 11-point scale and mandibular opening measured in millimetres, at baseline and again at three months, but only the banded forms of these variables were transcribed into the electronic dataset; the underlying integers and millimetre values remain on the paper clinical forms and could not be recovered for this analysis. Two things follow. The outcome had to be a dichotomized global rating rather than a change score, so neither change in pain intensity nor change in opening can be reported. And the more novel of the two findings, the difference in how pain presented, rests upon four ordered categories rather than upon the continuous scale from which they were derived. The direction of that finding is unlikely to be an artefact of banding, since the chewers were concentrated at the two extremes of a four-level scale, but its magnitude cannot be quantified and the bimodality itself might look different on the original scale. Recovering and entering the continuous values would be the single most valuable addition to this dataset, and we recommend it to any group attempting to replicate the work.

Symptom duration before presentation is the most consequential omission. It ranks among the strongest prognostic factors for conservative TMD therapy, it was recorded for the cohort as a whole, and it was not analyzed by exposure group. Chewers in this cohort were appreciably older than non-chewers, and older patients may plausibly have carried their symptoms for longer before seeking care, so symptom duration could confound the primary comparison in the same direction as age. Age accounted for the crude difference once entered into the model; whether symptom duration would have accounted for more cannot be determined from these data. This is a limitation on the primary comparison itself, not merely a gap in the baseline description.

Adherence is the fourth, and it matters here more than it would in most trials. The behavioural core of the intervention was a soft diet and the avoidance of parafunctional loading, and chewing qat for several hours a day is a direct contradiction of that advice. Chewers were therefore, by definition, partially non-adherent to the component of treatment that every patient received. Adherence was asked about at each visit but never captured as an analyzable variable, so the degree of that non-adherence cannot be quantified, nor can it be separated from any pharmacological or structural effect of the habit. Had chewers fared worse, behaviour and biology would have been indistinguishable as explanations. The outcome was null, which makes the question less pressing, but it leaves the study unable to say whether a chewer who genuinely followed the behavioural advice would have done better than one who did not.

Three further weaknesses deserve mention. The decision rule underlying the global rating was not specified numerically in advance, which limits reproducibility. A single examiner assessed both exposure and outcome without blinding, as discussed above. And every multivariable estimate presented here rests upon 13 events and is hypothesis-generating; the confidence intervals of the five-predictor model are wide enough to accommodate most clinically plausible effects in either direction.

Measurement was coarse in several further respects. Emotional stress and tooth grinding were single-item self-reports rather than validated Axis II instruments or the STAB framework [44–46], so the bruxism variable represents probable sleep bruxism at best. Qat exposure was self-reported and dichotomized at three sessions per week, without session duration or quantity, and with duration recorded only in bands that could not be linked to outcome. Drug doses, dosing frequency, treatment duration, splint specification and adverse events were held upon individual treatment records and never entered into the study database; none of them is reported here. Diagnosis, moreover, followed RDC/TMD rather than the current DC/TMD.

Residual confounding remains probable. Beyond symptom duration, tobacco smoking, shammah (smokeless tobacco) use and socioeconomic status beyond a three-level rating were not recorded. Qat chewing in Yemen is socially patterned, and each of these is plausibly associated with both the habit and the outcome, so the adjusted estimates should not be read as having removed confounding.

### Clinical implications

For clinicians practising where qat is widely chewed, the practical message is modest and, arguably, reassuring. Conservative, reversible management (education, self-care, short courses of medication and a splint where indicated) improved nine patients in ten, and habitual chewers benefited approximately as often as anyone else, within the limits of precision described above. Nothing within these data justifies withholding conservative therapy from chewers, escalating to irreversible treatment sooner, or advising a patient that qat use will prevent treatment from working. Counselling regarding the habit remains worthwhile; the case for it, however, rests upon the severity and structural findings rather than upon treatment response, and it accords with the INfORM/IADR emphasis upon conservative, reversible care and the avoidance of overtreatment [47–50].

## Conclusion

Among 148 Yemeni patients treated conservatively for TMD, 91.2% improved within three months, and no association was detected between habitual qat chewing and a poorer response (87.0% versus 93.6%, p = 0.23). That finding must be read for what it is. With power sufficient only for a difference of 16 percentage points or more, the study cannot exclude a clinically meaningful disadvantage to chewers, and the crude difference it did observe lost its apparent significance once the older age of the chewers was taken into account, in a model resting on 13 events that can only generate hypotheses. Pain also presented differently in chewers, clustering at both extremes rather than shifting uniformly upwards, and being more intense among those who had any. Whether that pattern reflects qat shaping the presentation of TMD rather than its response to treatment is a question these data raise and cannot answer. The practical implication is narrow but useful: there is no evidence here that would justify withholding conservative care from a patient who chews qat. Settling the wider question will require validated Axis II instruments, quantified exposure, blinded outcome assessment, measurement of symptom duration before presentation, and pain intensity and mandibular opening recorded as continuous variables at both visits.

## Supporting information

S1 Checklist STROBE checklist for cohort studies. (DOCX)

S1 Data De-identified individual-level dataset underlying all analyses reported in this manuscript (148 records; exposure, covariates and outcome). (XLSX)
