# Supplementary material for: Habitual qat chewing and short-term response to conservative management of temporomandibular disorders: A prospective cohort study in conflict-affected Yemen
Source: PLoS One. 2026 Nov 13;21(11):e0362744. doi: 10.1371/journal.pone.0362744 (PMC13766270; doi:10.1371/journal.pone.0362744)
Supplement: Supplementary file 1 [file pone.0362744.s004.pdf]

Response to Reviewers

Manuscript: “Habitual Qat Chewing and Short-Term Response to Conservative Management of Temporomandibular Disorders: A Prospective Cohort Study in Conflict-Affected Yemen”

(previously: “Association of Habitual Qat Chewing With Short-Term Clinical Improvement Following Conservative Management of Temporomandibular Disorders: A Prospective Cohort Study in Conflict-Affected Yemen”)

Corresponding author: Dr. Raji Mansoor Hajeb

Dear Editor,

We are grateful to both reviewers, and we owe the editor and the reviewers a frank statement before anything else.

Reviewer 2 wrote that “the risk ratio does not match your own counts” and asked us to recheck it. We did. Going back to the source SPSS file and to the doctoral thesis from which this cohort is drawn, we found that the recheck could not stop at the risk ratio. The Results section of the submitted version was not an accurate report of the dataset. The analysed sample was given as 140 (54 chewers, 86 non-chewers); the file contains 148 patients (54 chewers, 94 non-chewers). Thirty-two treatment failures were reported; the file contains 13. The sex distribution attributed to the two groups was wrong, and the source of that error is visible on inspection: “74.1% male” among chewers is the pain prevalence among chewers (40/54), transcribed into the wrong row. The reported logistic regression, the Spearman correlation and the duration–response figure cannot be reproduced from the data at all, and the variable for duration of qat chewing in years is not present in the analysis file.

We do not offer this as a defence. The errors were ours; they were introduced during manuscript preparation rather than during data collection, and the reviewers were right to find the numbers internally inconsistent. What we can do is correct them completely and make the correction verifiable, which is what this revision does.

The entire analysis has been rerun from the source file. Every number in the revised manuscript was re-derived programmatically from it. The de-identified individual-level dataset is supplied as S1 Data and the analysis code as S1 Text. Anyone can now run the code against the data and obtain the tables as printed.

The corrected analysis changes the paper’s conclusion. Improvement occurred in 47 of 54 chewers (87.0%) and 88 of 94 non-chewers (93.6%); the difference is 6.6 percentage points (95% CI −16.8 to 3.7), Fisher $p = 0.23$. The crude odds ratio of 0.47 attenuates to 0.92 after adjustment for age and sex, because chewers in this cohort were markedly older and age was the strongest predictor of non-improvement. The paper is therefore now a null result on its primary question, with a secondary finding that chewers presented with more severe pain. We have rewritten the title, abstract, results, discussion and conclusion accordingly, and we have removed the dose–response analysis rather than attempt to re-estimate it from data we do not have.

We recognise that the editor may wish to treat a correction of this magnitude as more than a routine revision, and we will cooperate with whatever process the journal considers appropriate.

Responses to Editor

Editor point 1: Required files

Four files are uploaded: this Response to Reviewers; a Revised Manuscript with Track Changes; a clean Manuscript; and supporting information (S1 Checklist, S1 Data, S1 Text analysis). File naming follows PLOS ONE conventions.

Editor point 2(a) and 2(b): Data sharing restrictions

There are no ethical or legal restrictions on sharing these data, and we withdraw the restricted statement made in the original submission form. It was made in error, out of an over-cautious reading of our ethics approval; the approval contains no such restriction and neither committee has imposed one. Sub-point (a) therefore does not apply.

Responding to sub-point (b): the minimal de-identified individual-level dataset necessary to replicate every finding is uploaded as S1 Data (148 records; exposure, all covariates and the outcome), together with the analysis code as S1 Text. The dataset contains no direct or indirect identifiers. Names, hospital record numbers, dates of birth, visit dates and all free-text clinical notes have been removed. Age is given in the five bands in which it was originally recorded, never as a date or an exact year. No combination of the retained variables identifies an individual within a cohort of 148 drawn from a large teaching-hospital catchment. Following the guidance the editor cited, we reviewed the file specifically for the risk of re-identification through variable combinations and are satisfied that none exists.

Editor point 3: Data Availability statement in the submission form

The submission-form statement has been replaced. It now reads: “All relevant data are within the manuscript and its Supporting Information files.” No exemption request is required, since the data are being made freely available.

Editor point 4: ORCID iD

The corresponding author’s ORCID iD is 0009-0001-5289-4494. It has been fetched and validated in Editorial Manager through “Update my Information”, and it appears on the manuscript title page.

Editor point 5: Ethics statement location

The ethics statement now appears once, in the Methods, under the heading “Ethics statement”. The duplicate statement that previously appeared in the Declarations section has been deleted. The statement names both approving bodies, the approval number and the date.

Editor point 6: Supporting Information captions

Captions for all Supporting Information files now appear at the end of the manuscript, after the reference list, under the heading “Supporting information”. Each file is cited where it is relevant: S1 Checklist in the Methods under study design, and S1 Data and S1 Text in the Methods under statistical analysis and again in the Data availability section.

Editor point 7: Reviewer-recommended citations

No reviewer required us to cite a specific publication of their own. Reviewer 2 pointed out that our own reference 23 contradicted a claim in our Introduction; we have engaged with that reference directly rather than added citations. Six references have been added, each needed to support new methodological or

substantive content: the original RDC/TMD publication [45], a 2026 systematic review and meta-analysis of khat and TMD [46], the STROBE statement [47], Firth's penalised likelihood method [48], the events-per-variable simulation study [49] and a standard source for the Numeric Rating Scale [50].

Responses to Reviewer #1

We thank Reviewer 1 for a careful reading and for identifying the absence of quantified baseline severity as the central weakness. That point turned out to be partly answerable from our records, as set out below.

Major point 1: RDC/TMD rather than DC/TMD

Accepted, and now both justified and acknowledged as a limitation. The Methods now state explicitly that RDC/TMD Axis I was used and cite the original RDC/TMD publication [45] rather than the DC/TMD paper. The reason is given as well. The examination protocol, data collection forms and Arabic-language interview schedule for the parent doctoral project were built and piloted against the RDC/TMD before recruitment opened, and the examiner was calibrated against that instrument. Changing instrument in the middle of a 25-month recruitment period would have made the diagnoses non-comparable across the cohort. We accept without reservation that the DC/TMD had been available for four years by 2018 and is the current standard, and this now appears in the Limitations.

Major point 2: No quantified baseline severity

This is where our original manuscript did the study a disservice. Baseline severity was in fact quantified. Pain intensity was recorded at the first and last visits on a written 11-point Numeric Rating Scale, reported in the conventional bands (0; 1–3 mild; 4–6 moderate; 7–10 severe). Mandibular range of motion was measured in millimetres with a ruler between the incisal edges, with the vertical incisal overlap added: pain-free unassisted opening, maximum painful unassisted opening and maximum assisted opening. Both measures were part of the parent protocol, and both are in the source data. The submitted manuscript simply failed to report them.

They are now reported, and they carry the paper's most interesting finding. The two groups did not enter the study with comparable severity, contrary to what the previous version asserted. Not one qat chewer reported mild pain, whereas 32 of 94 non-chewers (34.0%) did; 13 chewers (24.1%) reported severe pain against 8 non-chewers (8.5%); across the four bands $p < 0.001$. Restricting to patients reporting any pain, 38 of 40 chewers (95.0%) rated it moderate or severe against 52 of 82 non-chewers (63.4%). This appears in Table 2 and is discussed as a substantive result.

One limitation remains and we state it plainly: the follow-up NRS scores and post-treatment opening measurements were recorded on the clinical forms but were not transcribed into the analysis dataset, so we cannot compute change scores. We have not attempted to reconstruct them.

Major point 3: Outcome measures not operationally defined

Accepted, and the previous description was worse than vague: it was wrong. Two separate endpoints, subjective and objective, were described in the Methods and a composite requiring both was introduced in the Results. Neither exists. A single outcome was recorded: at the three-month visit the examiner recorded the patient's global rating of change on the four-point scale used throughout the parent protocol; much better, better, improved, not improved, taking account of the three presenting complaints. For analysis this was dichotomised as improved (the first three) versus not improved. That is the operational rule, it is now

stated in the Methods, and it is the only outcome in the dataset. We have removed all reference to separate subjective and objective endpoints and to a composite.

We also accept the corollary: because the rule rests on a patient's global judgement rather than on a numerical threshold specified in advance, it is less reproducible than a defined change in NRS or in millimetres of opening would have been. That is now listed among the limitations rather than glossed over.

Major point 4: Blinding and reliability

Both accepted. The Methods now carry a short section headed "Blinding". It states that the examiner who recorded qat chewing status at baseline also recorded the outcome at three months, that blinding was therefore not achieved, and that in a setting where qat use is disclosed openly and its signs are visible it could not have been achieved by a single-examiner design. We also give the likely direction of the bias: towards the departmental expectation that chewers do worse, which would exaggerate a disadvantage among chewers rather than mask one. The result was null, so the bias does not explain what we found. It would have mattered a great deal had the result been positive. We have also stopped describing the outcome as objective; it is called a patient-reported global rating throughout.

On reliability: you are right that with one examiner the relevant quantity is intra-examiner agreement. The manuscript now says intra-examiner and the phrase "inter-rater reliability" has been removed.

Major point 5: Sex imbalance

This concern rested on figures that were wrong in the submitted manuscript, and we are sorry to have sent the reviewer chasing an artefact. The true distribution is 20 of 54 chewers male (37.0%) and 25 of 94 non-chewers male (26.6%), p = 0.20; a 10-point difference, not 40. The 74.1%/34.9% split was the pain prevalence among chewers transcribed into the sex row.

We have nonetheless done the analyses the reviewer asked for, because they are worth having. Variance inflation factors in the full model run from 1.05 to 1.31, so sex and qat chewing are not collinear in these data. Sex-stratified estimates show nothing in either stratum: among men 17 of 20 chewers (85.0%) versus 22 of 25 non-chewers (88.0%) improved (p = 1.00); among women 30 of 34 (88.2%) versus 66 of 69 (95.7%) (p = 0.22). The Mantel–Haenszel odds ratio stratified by sex is 0.50, close to the crude estimate. All of this is now in the Results.

Major point 6: Overfitting and power

Accepted, and the issue is in fact more consequential than the reviewer could have discerned from the submitted manuscript: the true number of non-improvement events is 13, not 32, so a five-predictor model carries 2.6 events per variable rather than 6.4. A conventional maximum-likelihood logistic regression on these data is not defensible. All multivariable models are now fitted by Firth penalised likelihood [48], with profile penalised-likelihood confidence intervals and penalised likelihood-ratio p-values, and every model is labelled exploratory in the Methods, the Results and the Conclusion. We report three: unadjusted; a minimal model with age and sex (4.3 events per variable); and the full five-predictor model retained for comparability with what was originally specified. C-statistics are given (0.60, 0.73, 0.77) with the caveat that they are unstable at this event count.

A power discussion has been added to the Methods. With 54 chewers, 94 non-chewers and a 93.6% improvement rate among non-chewers, the study had 80% power to detect an absolute difference of 16.1

percentage points or larger. Power to detect the 6.6-point difference observed was 0.26. We say in the Conclusion that this is an absence of evidence rather than evidence of absence.

Minor point 1: Dose–response figure

The figure has been removed and so has the Spearman analysis. The reviewer's instinct that a smooth curve over 54 subjects was misleading was correct, but the underlying problem is more basic: duration of qat chewing was recorded only in three bands (under 10 years, 10–20 years, over 20 years) and was not carried into the analysis dataset alongside the outcome. No dose–response analysis can be performed on the data we hold. We report the band distribution descriptively (17, 23 and 14 patients respectively) and state in the Methods why no dose–response analysis is presented.

Minor point 2: CYP speculation

Agreed and rewritten. The Discussion now states that we measured no pharmacokinetic and no neuromuscular variable, assayed no cathinone and measured no drug levels, and that the CYP interaction is a hypothesis our setting raises rather than one our findings support. We have also pointed out that our data are mildly unhelpful to that hypothesis: chewers and non-chewers received near-identical pharmacotherapy and had near-identical outcomes.

Minor point 3: Typographical errors

The truncated "ong-term", the incomplete funding sentence and the double period in the Table 4 title have all gone, along with the rest of the Results and Discussion in which they sat. The full manuscript has been proofread.

Minor point 4: Reference numbering and the STAB citation

Corrected, and see our fuller answer to Reviewer 2 under References below. The Ethiopian undernutrition meta-analysis is now cited as [42], matching its position in the list. The STAB framework is cited as [43,44], both of which exist; the previous "[44,45]" pointed to a reference 45 that did not. The whole text-to-list mapping has been checked citation by citation.

Minor point 5: STROBE checklist

Added as S1 Checklist, completed against the cohort-study version, with page numbers keyed to the clean manuscript. The STROBE statement is cited in the Methods [47].

Minor point 6: Ethics document title and resubmission history

A fair question and we should have addressed it unprompted. The ethics approval was granted to the parent doctoral protocol, "Evaluation of the outcome of conservative management of temporomandibular disorders among patients attending Sana'a University and Al-Kuwait Teaching Hospital", which is why the approval document carries a different title from the manuscript. The present paper reports a comparison planned within that protocol, and its title describes that comparison rather than the protocol. This is now explained in the ethics statement in the Methods, which gives the protocol title alongside the approval number and date. We confirm that this cohort has not been published elsewhere; the parent thesis is a university deposit, not a publication.

Responses to Reviewer #2

We thank Reviewer 2 for a review of unusual thoroughness. The judgement that the problems were "fixable with information you probably already hold" was correct in most cases, and following the comments in order was what led us to re-open the source file and find the errors described at the start of this letter.

Introduction

The claim that no prospective study exists is contradicted by reference 23

Accepted; the claim was too broad. Bakhadher et al. [23] is indeed prospective. It followed 240 khat chewers in Aden between 2013 and 2015 with a pilot-tested questionnaire and reported the prevalence of self-reported TMJ disorder (34.2%). It enrolled no comparison group of non-chewers, made no clinical Axis I diagnosis, delivered no treatment and collected no outcome data. The Introduction now describes that study specifically and states how ours differs: consecutive treatment-seeking patients with a clinical RDC/TMD Axis I diagnosis, both chewers and non-chewers, managed conservatively and re-examined at three months.

We have also strengthened the framing with evidence that post-dates our submission. A systematic review and meta-analysis published in 2026 pooled 13 observational studies and 3,526 participants and found chewers about two and a half times more likely to have TMD (OR 2.48, 95% CI 1.20–5.09), while noting that the evidence is of low quality and heterogeneous [46]. That review identified no study of treatment response in chewers, which is a more defensible statement of our novelty than the one we made, and it is now the one we make.

The aim mentions "potential confounders" without naming them

Named. The final paragraph of the Introduction now states that the comparison was planned with adjustment for age, sex, self-reported emotional stress and self-reported tooth grinding, being the four baseline covariates judged before analysis most likely to confound the association. These are the four variables that appear in the full model in Table 5, so the reader can check the model against the stated intention.

Methods

Is this really an observational study?

Yes, and we accept that the previous wording did not make that clear. The Methods now state that no aspect of management was assigned by the investigators. Which drug, at what dose, and whether a splint was made were decisions taken by the treating oral medicine specialist on clinical grounds, under the department's standing TMD protocol, exactly as they would have been taken outside the study. The investigators recorded what was prescribed. They did not determine it. This was routine departmental care, the study was observational, and prospective registration was accordingly not required. We are content for the editorial office to review that judgement.

Outcome definitions

This was the most serious of the reviewer's methodological points and we accept it in full. Please see our response to Reviewer 1's major point 3 for the operational rule, which is now in the Methods: a single four-point global rating of change (much better / better / improved / not improved) recorded at the three-month visit and dichotomised for analysis. The phrases "substantial reduction", "observable functional improvement" and "pain-free maximum interincisal opening" as endpoint criteria have been deleted, because they described endpoints that were never recorded.

On the instrument and the threshold: mandibular opening was measured with a millimetre ruler between the maxillary and mandibular central incisal edges with the vertical incisal overlap added, and the 40 mm threshold used in the parent protocol for limited opening is now stated. But we must be straightforward with the reviewer; opening in millimeters was recorded at baseline and re-measured at three months, yet only the baseline values were transcribed into the analysis dataset. The outcome variable that exists is the global rating, not a millimetre change. We would rather say this than construct a threshold rule after the fact.

The endpoint analysed is not the one specified

Correct, and the explanation is that neither endpoint specification was accurate. There were never two primary outcomes and there was never a composite. A single global outcome was recorded and a single global outcome is now reported. Nothing was pre-specified as a composite because no composite existed; the wording in the previous Results was an error, not a change of plan. The Methods now describe the one outcome and the Results report it.

One examiner assessed both exposure and outcome, unblinded

Accepted; see the response to Reviewer 1's major point 4. A dedicated "Blinding" subsection now states this, discusses the likely direction of the bias, and the outcome has been relabelled as examiner-recorded and patient-reported rather than objective.

"Inter-rater reliability (Kappa > 0.85)"

Accepted on the terminology: the figure is intra-examiner agreement, and it is now described as such throughout. On the detail we must be straightforward. Calibration against the RDC/TMD protocol was performed before recruitment, and agreement on repeat examination exceeded kappa 0.85 for every dichotomous examination item; the Methods now name those items explicitly. The calibration worksheet recording the number of patients re-examined, the interval between examinations and the item-level kappa with its confidence interval has not survived in retrievable form, and those figures cannot therefore be reported. We would rather state the summary we can vouch for and acknowledge the missing detail than supply numbers we cannot document.

Diagnostic criteria and citation do not match; report Axis I distribution by group

Both done. The instrument is RDC/TMD Axis I, now cited to the original publication [45]; the DC/TMD reference [1] is retained only where the DC/TMD itself is discussed. The justification and the acknowledgement of the limitation are given above.

The Axis I distribution by group is now in Table 2, and the reviewer's concern was well founded; the groups differ. Disc displacements were more frequent among chewers (35.2% versus 19.1%, $p = 0.048$) and masticatory muscle disorders less frequent (42.6% versus 62.8%, $p = 0.025$); arthralgia and osteoarthritis were similarly distributed (63.0% versus 78.7%, $p = 0.054$). Because 82 patients met criteria for more than one group, the groups are reported non-exclusively, and we have not attempted to force each patient into a single category.

Treatment was not uniform and we are not told who received what

A table of treatment components by group is now provided as Table 3, with the doses as prescribed. The reviewer's hypothesis is not supported: treatment was distributed almost identically. Any pharmacotherapy 77.8% of chewers versus 85.1% of non-chewers ($p = 0.27$); NSAID 74.1% versus 83.0% ($p = 0.21$); anxiolytic 53.7% versus 61.7% ($p = 0.39$); amitriptyline 20.4% versus 22.3% ($p = 0.84$); stabilisation splint 24.1% versus 25.5% ($p = 1.00$). Confounding of the exposure–outcome comparison by differential treatment is therefore unlikely, and we now say so in the Results rather than leaving it to be assumed.

On doses and on the splint specification we owe the reviewer a direct answer, and it is not the answer we would prefer to give. We went back to the source documents; the analysis dataset and the parent doctoral thesis, and neither records a dose, a dosing frequency or an intended duration for any of the three drugs. Neither records the material, arch, occlusal coverage, wear instructions or review schedule of the appliance. Those details were written on individual treatment records at the time of care. They were never carried into the study database, and the records are not available to us in a form that would let us report them for the cohort.

The query about amitriptyline turned out to be the thread worth pulling. The figure of 20 mg cannot be traced to any study document. Every milligram figure in the parent thesis sits in its literature review and belongs to a trial by other investigators, naproxen 500 mg twice daily, diclofenac 50 mg three times daily, amitriptyline 25 to 75 mg daily, diazepam 10 mg rising to a maximum of 20 mg — and none of them describes what was prescribed here. The doses given for naproxen and diazepam in the previous version are equally untraceable. Every dose has therefore been removed from the Methods and from Table 3 rather than replaced with a plausible substitute, and the Methods state without euphemism that doses cannot be reported and why. Table 3 gives the agents and the proportion of each group receiving them, which is what the source documents support.

We recognise that this is a thinner answer than a table of doses would have been. It is the accurate one. Having already had to correct a Results section built on figures that could not be traced to source, we are not willing to introduce new ones into the Methods. We would add that the thesis also names diclofenac sodium alongside naproxen as the NSAIDs used; the previous version mentioned only naproxen, and Table 3 and the Methods now name both.

Adherence was monitored but never reported

We have to concede this one rather than answer it. Adherence was asked about at each follow-up visit, self-care advice, medication, splint wear, but the answers were never captured as an analysable variable, and we cannot report them by group. The Methods and the Limitations now say exactly that, together with the reason it matters, which is the reviewer's own point about differential adherence, addressed below.

The exposure measure is coarse

Accepted. The Methods now state what was and was not recorded: chewing status by structured interview with a threshold of three or more sessions per week; duration in three bands; habitual chewing side as left, right or bilateral. Session duration and quantity chewed were not recorded. No participant reported reducing or stopping qat use during the three months. The reviewer is right that chewing side is central to the unilateral-loading mechanism argued in the Discussion. The variable exists in the parent dataset, but it was not linked to the outcome in the analysis file. Rather than present a partial analysis, we have limited the mechanistic claims accordingly and named the omission in the Limitations.

Several likely confounders were not measured

All four are now named in the Limitations: tobacco smoking, shammah use, socioeconomic status beyond a three-level rating, and the duration of TMD symptoms before presentation. We agree with the reviewer that the last of these is the most damaging omission, since duration of symptoms is among the strongest prognostic factors for conservative therapy. It was recorded for the cohort as a whole in the parent protocol but was not analysed by exposure group, and we have not gone back and constructed the comparison post hoc.

Participant flow

Added, as Fig 1 and in the Results. Of 156 patients screened between October 2018 and October 2020, 8 did not meet the eligibility criteria and 148 were enrolled. All 148 attended the three-month visit; there was no loss to follow-up in either group and no missing outcome data. The exclusion criteria are stated in full in the Methods, although the screening log does not allow the eight exclusions to be apportioned between them. The reviewer was right to suspect differential attrition over 25 months in a difficult setting, as it happens there was none, but that could not be judged from the previous version, and it can now.

Safety paragraph for the pharmacological regimen

A safety paragraph has been added, and it says only what can be defended. Adverse events were not collected as a study variable. Patients were reviewed at three weeks, six weeks and three months, and an adverse effect reported at any of those visits would have been managed clinically and entered in the case record, but no structured safety information was extracted for analysis. No adverse-event rate can be given. We have been careful not to imply, by saying nothing, that none occurred.

The reviewer is right that this matters. The regimen included a benzodiazepine and a tricyclic antidepressant, and a paper reporting neither exposure duration nor discontinuation is thin on the safety side. The Methods say so, the Limitations say so again, and we name the variables a future study in this department should capture: duration of exposure to each agent, sedation, and discontinuation for adverse effect.

Results

Report subjective and objective outcomes separately

We cannot, and the reason is the one given above: separate endpoints were never recorded. Both the Methods specification and the Abstract claim in the previous version were wrong. A single global outcome exists, and it is reported.

The risk ratio does not match your own counts

It did not, and following that thread is what uncovered the wider problem set out at the start of this letter. The corrected figures are these. Improvement in 47 of 54 chewers (87.0%) and 88 of 94 non-chewers (93.6%); risk difference −6.6 percentage points (95% CI −16.8 to 3.7); risk ratio 0.93 (95% CI 0.83–1.04) by the Katz logarithmic method, now named in the Methods; odds ratio 0.47 (95% CI 0.15–1.42); Fisher's exact $p = 0.23$. We are grateful for the check.

The regression model is probably overfitted

It was, by more than the reviewer estimated. See the response to Reviewer 1's major point 6: 13 events, Firth penalised estimation throughout, profile penalised-likelihood intervals, C-statistics reported, and the exploratory framing now carried consistently through the Methods, Results and Conclusion as requested.

Sex is almost collinear with the exposure

It is not, once the sex figures are correct: 37.0% versus 26.6% male, p = 0.20. Variance inflation factors are 1.05 to 1.31. We have nonetheless added both analyses the reviewer asked for, sex-stratified estimates and a Mantel–Haenszel odds ratio stratified by sex, and both are reported in the Results.

The dose–response analysis needs rethinking

Removed in full, for the reason given under Reviewer 1's minor point 1. The reviewer raised three sub-points and each is well taken. We cannot state the n for the Spearman correlation, because the correlation cannot be reproduced from the data at all. The reviewer is right on the method too: a rank correlation between a continuous exposure and a binary outcome is the wrong test, and entering non-chewers as zero would merely restate the group difference. As for the 58% and 78% figures, they have no numerators or denominators anywhere in the dataset — which is also why the missing 10–20 year band could not be supplied. Duration exists only as three bands, unlinked to outcome. We report the band counts descriptively and present no analysis.

Some participants apparently had no pain at baseline

A good catch, and it exposed a data-consistency issue we have now disclosed. Twenty-five patients (16.9%) had an NRS band of 0 at baseline: 16 chewers (29.6%) and 9 non-chewers (9.6%). They were enrolled because they presented with joint sounds or limitation of opening rather than pain — the eligibility criterion was at least one TMD sign or symptom, not pain specifically. Their global rating of change was made against whichever complaints they presented with, which is how the four-point scale was applied throughout.

In checking this we found seven records (4.7%) internally discordant between the binary pain item and the NRS band: three coded as having pain were assigned band 0, and four coded as pain-free were assigned a mild or moderate band. We have left these records unaltered, reported both variables as recorded, disclosed the discordance in the Methods, and run a sensitivity analysis excluding the seven. It changes nothing: 45 of 52 chewers (86.5%) versus 84 of 89 non-chewers (94.4%) improved, Fisher p = 0.13.

Discussion and Conclusion

The Discussion is one paragraph running to two pages

Restructured into the sections the reviewer specified: Principal findings; Comparison with previous work; Mechanisms and what our data can and cannot say about them; Competing explanations; The conflict setting; Clinical implications; Strengths and limitations.

The language is causal where the design is not

Rewritten throughout in associational terms. "Appears to reduce the short-term effectiveness" is gone, as is "support a combined neuromuscular and pharmacokinetic explanation"; the latter was indefensible given that we measured no neuromuscular and no pharmacokinetic variable. The CYP interaction is now presented as a hypothesis the setting raises, and we note that our own data sit slightly against it. The title has been changed from "Association of … With … Improvement" to "Habitual Qat Chewing and Short-Term Response to …" for the same reason.

The most obvious competing explanation is not addressed

Now addressed directly, under "Competing explanations". Continuing to chew qat for several hours a day is itself a departure from the soft-diet and reduced-loading advice every patient received. Had chewers done worse, differential adherence to the behavioural component would have been at least as good an explanation as any biological one. We cannot argue against it with adherence data, because those data were not captured in analysable form, and we say so. What we can say is that the outcome was null, which weakens the competing explanation along with the primary hypothesis. We have not claimed more than that.

The Limitations are honest but incomplete

All four additions have been made: the unblinded single assessor, participant flow (now supplied rather than merely acknowledged), the unmeasured confounders listed above, and the low events-per-variable ratio. We have added several the reviewer did not raise. The absence of change scores, the unspecified decision rule behind the global rating, the coarse exposure measure, and a study powered only for a difference of 16 percentage points or more.

COVID-19 overlapped the end of recruitment

Now stated. The pandemic, together with the security situation, limited the number of patients who could be reached and contributed to the modest sample size. Follow-up completeness was not affected: an outcome was recorded at three months for all 148 enrolled patients. Whether recruitment was formally suspended at any point during 2020, and for how long, is flagged in the manuscript for completion from the departmental record.

References and language

Citation numbering is out of step with the list

Corrected and checked in full. The reviewer was right that the offset ran further than the two instances identified. Every in-text citation has been mapped against the reference list one by one. The Ethiopian undernutrition meta-analysis is cited as [42], its actual position; the STAB framework is cited as [43,44], both of which exist, replacing "[44,45]"; and the six new references occupy positions 45 to 50, appended rather than interleaved so that no existing number shifts.

Reference 36 looks misplaced

It was. Abdulkader, "The forgotten war", has been removed from the cluster supporting psychological factors in TMD, which now reads [31–35], and moved to the Discussion paragraph on the conflict setting, where it sits with [37,38].

Typographical recheck needed for the whole manuscript

Done. Much of the text has been rewritten, and what remains has been proofread.

"Qat" and "khat" used interchangeably

The equivalence is now noted once, at first mention in the Introduction, and "qat" is used consistently thereafter except in the titles of cited works, where the original spelling is retained.

Ethics, data availability and reporting

The data availability statement contradicts the manuscript

Resolved. S1 Data holds the de-identified individual-level dataset behind every analysis, not aggregate data. The submission-form statement now reads "All relevant data are within the manuscript and its Supporting Information files." We have also added S1 Text, the analysis code, so that the tables can be reproduced from the file rather than merely checked against it. Given what this revision has had to correct, reproducibility is the least we can offer.

The two ethics statements differ

Made identical. Both the manuscript and the submission form now name both approving bodies with number and date. They are the Medical Research Ethics Committee, Faculty of Dentistry, Sana'a University (Approval No. 166, 15 September 2018), and the Research Committee, Faculty of Dentistry, National Ribat University, Khartoum (approved 2018; that committee issues no reference number). The ethics statement appears once, in the Methods, as the editor requires.

Please supply the STROBE checklist

Supplied as S1 Checklist, completed against the cohort-study version with page numbers keyed to the clean manuscript. The reviewer's observation that several of the gaps map onto STROBE items proved accurate: items 6 (participants and flow), 8 (measurement), 12 (statistical methods), 13 (participant numbers) and 22 (funding) all required substantive additions rather than page numbers.

Summary of the principal changes

1. Title changed to remove the causal implication and to reflect a null primary finding.

2. Entire analysis rerun from the source SPSS file; N corrected from 140 to 148, non-chewers from 86 to 94, non-improvement events from 32 to 13, and the sex distribution corrected.

3. Primary result changed: improvement 87.0% in chewers versus 93.6% in non-chewers, Fisher $p = 0.23$. Abstract, Results, Discussion and Conclusion rewritten accordingly.

4. Crude association shown to be confounded by age; adjusted odds ratio 0.92 (95% CI 0.26–3.29).

5. Baseline pain severity (NRS) and mandibular opening in millimetres reported for the first time; chewers presented with more severe pain ($p < 0.001$).

6. Outcome definition corrected to the single global rating that was recorded; the fictitious composite removed.

7. All multivariable models refitted with Firth penalised likelihood and labelled exploratory; power analysis added.

8. Dose–response analysis and Figure 2 removed as unsupported by the data.

9. Sex-stratified analysis, Mantel–Haenszel estimate and variance inflation factors added.

10. Participant flow (Fig 1), treatment components by group (Table 3), Axis I distribution by group (Table 2), blinding subsection and safety paragraph added.

11. Discussion restructured into seven sections; causal language removed; the adherence counter-explanation addressed directly.

12. Reference numbering corrected throughout; reference 36 repositioned; six references added.

13. S1 Checklist (STROBE), S1 Data (de-identified individual-level dataset) and S1 Text (analysis code) supplied; data availability statement corrected.

14. Ethics statement consolidated in the Methods and made identical to the submission form.

15. Manuscript brought into line with PLOS ONE style throughout. The title is in sentence case and a short title has been added. The abstract has been rewritten as a single unstructured paragraph of 297 words, within the 300-word limit and free of citations. Sections run Introduction, Materials and Methods, Results, Discussion, Conclusion, Acknowledgements, Author contributions, Funding, Competing interests, Data availability, References, Supporting information. Figure captions now sit immediately after the paragraph in which each figure is first cited, and tables directly after the paragraph citing them; figures are supplied separately as 300 dpi TIFF files. Author contributions are restated using the CRediT taxonomy. The full reference list has been reformatted to PLOS Vancouver style, with author lists truncated to six followed by et al., abbreviated journal titles, and DOIs where available.

16. Four entries in the original reference list were incomplete or contained transcription errors. These have been corrected where the correct form is certain, and flagged in the list for the authors to complete against the source publications where it is not, so that no unverified detail enters the paper.

17. All drug doses removed from the Methods and from Table 3. Neither the analysis dataset nor the parent doctoral thesis records a dose, dosing frequency or intended duration for naproxen, diclofenac, diazepam or amitriptyline, and neither records the specification of the stabilisation splint. The Methods state this openly and report the agents and the proportion of each group receiving them. Diclofenac sodium is now named alongside naproxen, as the thesis records.

18. Safety paragraph added, stating that adverse events were not collected as a study variable, so that no adverse-event rate is given and none is implied by silence. The Limitations name the safety variables a future study should record.

19. The manuscript has been rewritten end to end for register and flow. Passages that argued with the previous review — references to “the previous version”, to “the reviewer”, and to errors “corrected”, have been removed from the paper and are itemised here instead, where they belong. Sections were merged where they had fragmented (blinding now sits within the examination section, where the examiner is described), the single 300-word Limitations paragraph was restructured into four organised by threat, and the Discussion follows principal findings, comparison with previous work, mechanisms, alternative explanations, setting, limitations and clinical implications.

20. Every in-text citation was audited against the title and scope of the work cited, and nine mismatches were corrected. Reference 6 (Greene, a care guideline) had been cited for aetiology and now supports the sentence on conservative management. References 9 and 10, both concerned with post-traumatic stress and TMD, had been cited for the prevalence of psychological morbidity in the Yemeni civilian population and now support the TMD link instead, that population claim resting on references 11 and 37. The bracket [14-18] had swept two imaging studies and a prevalence survey into a claim about cathinone pharmacology; the pharmacology now cites 18 and 25, the prevalence of the habit cites 14 and 17, and the imaging remains with 15 and 16. Reference 25 (khat and appetite) no longer supports a claim about chewing biomechanics, reference 27 (khat pharmacokinetics) has moved from a pharmacodynamic claim to the cytochrome P450 sentence, reference 29 (oral symptoms in khat users) has moved from that sentence to the oral-disease

burden, and reference 38 (awake bruxism and psychological scores in Italian adults) no longer supports statements about war in Yemen and now sits with the bruxism-measurement limitation. All 50 references are cited, and no citation points outside the list.

21. Reference details for the works added at revision were checked against PubMed and corrected where they differed: the full title of reference 50 (Hawker et al., Arthritis Care Res 2011;63 Suppl 11:S240-52), the spelling of Gøtzsche and the page range in reference 47 (Lancet 2007;370(9596):1453-7), and the page ranges of references 45 and 49. Two entries that could not be verified — reference 9, in a journal PubMed does not index, and reference 11, a report without publisher details — carry an explicit note asking the authors to confirm them against the originals rather than a reconstructed citation.

22. The ethics statement now confirms that the 18 participants in the 10-20 year age band were minors under Yemeni law, that written consent was obtained from a parent or legal guardian for each of them in addition to the participant's own assent, and that the ethics committee approved their inclusion. The corresponding author's ORCID iD has been validated and appears on the title page.

23. Reference 11 has been replaced with the full citation of the source: Sana'a Center for Strategic Studies, Columbia Law School Human Rights Clinic and Columbia University Mailman School of Public Health. The impact of war on mental health in Yemen: a neglected crisis. Sana'a: Sana'a Center for Strategic Studies; 2017. The earlier entry named the wrong authors and lacked publisher details.

24. The Discussion subheadings introduced at the first revision have been removed, and the section now runs as continuous prose under Discussion, Strengths and limitations, and Clinical implications. The structure the reviewer requested is nonetheless retained in full: principal findings, comparison with previous work, mechanisms, alternative explanations, the conflict setting, limitations and clinical implications each occupy their own paragraph or paragraphs, in that order. Should the editor prefer explicit signposting, the subheadings can be restored without altering a word of the text.

25. Em dashes have been removed throughout in favour of commas, colons and parentheses. The punctuation of the affected sentences was reset accordingly, so that the appositive lists in the exclusion criteria, the reliability items, the sociodemographic variables, the four-point outcome scale and the clinical-implications sentence each read correctly.

We thank the editor and both reviewers for the time they have given this manuscript. The paper that results is a smaller claim than the one we submitted, but it is one the data support.

On behalf of all authors,

Dr. Raji Mansoor Hajeb
