# Supplementary material for: Habitual qat chewing and short-term response to conservative management of temporomandibular disorders: A prospective cohort study in conflict-affected Yemen
Source: PLoS One. 2026 Nov 13;21(11):e0362744. doi: 10.1371/journal.pone.0362744 (PMC13766270; doi:10.1371/journal.pone.0362744)
Supplement: Supplementary file 2 [file pone.0362744.s005.pdf]

Response to Reviewers

Manuscript: "Habitual qat chewing and short-term response to conservative management of temporomandibular disorders: a prospective cohort study in conflict-affected Yemen"

Corresponding author: Dr. Raji Mansoor Hajeb

We thank both reviewers for their careful re-evaluation of the manuscript. Reviewer 1's second point prompted a re-examination of the dataset, which led us to revise one of the paper's two principal claims. We address this correction first, as it bears on the responses that follow.

A correction arising from Reviewer 1, point 2

Reviewer 1 asked why the continuous baseline measures cannot be analysed, and whether they might be incorporated. In addressing this question, we found that the severity finding had been described incorrectly.

The previous version stated that qat chewers presented with greater pain severity. Re-analysis of the banded Numeric Rating Scale variable as ordinal data, rather than as four unordered categories, does not support this statement. Chewers were over-represented at both ends of the scale: 16 of 54 (29.6%) reported no pain at all against 9 of 94 non-chewers (9.6%), and 13 (24.1%) reported severe pain against 8 (8.5%), whereas no chewer fell within the mild band that contained 32 of 94 non-chewers (34.0%). The distributions therefore differ in shape, not in location. A Wilcoxon rank-sum test across the ordered bands returned $p = 0.22$, the median band is moderate in both groups, and the odds ratio reverses direction across the three cut-points, from 0.25 for any pain versus none, through 1.84 for moderate-or-severe versus mild-or-none, to 3.41 for severe versus the rest. The proportional-odds assumption therefore does not hold, and the four-band chi-square of $p < 0.001$ that we previously reported reflects that change in shape rather than any overall elevation of severity.

The finding that remains is narrower and more precisely specified: among the 122 patients who reported any pain, that pain was moderate or severe in 38 of 40 chewers (95.0%) against 52 of 82 non-chewers (63.4%), $p < 0.001$. This is a conditional contrast, and the manuscript now describes it as such wherever the finding appears: in the abstract, Results, Discussion and Conclusion. We have added the rank-sum test and the cut-point odds ratios to the Methods and

Results, and a note to Table 2. We are grateful to the reviewer for a question that has made the paper more accurate, if less straightforward.

Reviewer #1

We thank the reviewer for the assessment of the previous revision and for six specific points, each of which has led to a change in the text.

1. Power and interpretation

We accept this point in full and have applied it in four sections of the manuscript. The abstract now closes: "Powered only for a difference of 16 percentage points or more, the study cannot exclude a clinically meaningful disadvantage to chewers; all adjusted estimates rest on 13 events and are hypothesis-generating." The Results no longer state only that the difference is compatible with chance; the sentence now states that the data are compatible with no difference between the groups, that the interval is wide, and that a disadvantage to chewers of as much as 17 percentage points is equally compatible with them. The Discussion opens its account of the principal findings by saying that the study is unable to exclude a clinically meaningful disadvantage and that the appropriate reading is an absence of evidence rather than evidence of absence. The Conclusion makes the same point at the outset.

Regarding the labelling of the models, the term "exploratory" has been replaced throughout by "hypothesis-generating", which conveys the intended meaning more clearly; the term now appears in the Methods, at the head of the multivariable Results, in the legend to Fig 2, in the title and footnote of Table 5, and twice in the Discussion. The Methods add a sentence that no estimate drawn from these models should be read as an effect size, and the Results section introducing them states that they are presented to show the direction in which adjustment moves the crude estimate, not to quantify an effect.

2. Baseline severity data

This was the most consequential point and has led to the largest revision, described at the beginning of this letter. On the specific question of why the continuous measures cannot be analysed, the Methods now explain the reason rather than simply stating the constraint. Pain intensity was rated by the patient on an 11-point scale, but the integer score was transcribed into the study database only after being collapsed into the conventional bands; the underlying 0-10

value was retained solely on the paper clinical form. Mandibular opening was handled identically, the millimetre measurements being entered as the threshold categories used in the parent protocol rather than as continuous values. The electronic dataset therefore contains ordered categories alone. This is a transcription decision taken at the time of the parent doctoral project, not a loss of data, but the paper forms are not available to us in a form that would permit retrospective entry for 148 patients.

Within these constraints, we have treated the bands as ordinal rather than nominal variables. This re-analysis produced the correction described above, which would not otherwise have been identified. The Limitations section now includes a paragraph on this issue as the second of four limitations bearing directly on interpretation, stating that the direction of the severity finding is unlikely to be an artefact of banding, since chewers were concentrated at the two extremes of a four-level scale, but that its magnitude cannot be quantified and the bimodality itself might look different on the original scale. We identify recovery of the continuous values as the most valuable addition that any replication could make.

3. Unmeasured confounders, particularly symptom duration

We agree. Symptom duration is now addressed in a dedicated paragraph in the Limitations, placed third among the four that bear on interpretation, and it is framed as a limitation on the primary comparison rather than on the baseline description. The paragraph develops the argument implied by the reviewer's comment: chewers in this cohort were appreciably older than non-chewers, older patients may plausibly have carried their symptoms for longer before presenting, and symptom duration could therefore confound the primary comparison in the same direction as age. Since age accounted for the crude difference once entered into the model, the possibility that symptom duration would have accounted for more cannot be dismissed, and the present data cannot resolve it. The remaining unmeasured confounders are retained in a separate, shorter paragraph, with the observation that qat chewing in Yemen is socially patterned and that the adjusted estimates should not be read as having removed confounding.

4. Adherence

We agree, and adherence is now presented as the fourth limitation bearing on interpretation. The paragraph now states explicitly that the behavioural core of the intervention was a soft diet and the avoidance of parafunctional loading, chewing qat for several hours a day directly contradicts

that advice, and chewers were therefore by definition partially non-adherent to the one component every patient received. Because adherence was never captured as an analysable variable, that non-adherence cannot be quantified and cannot be separated from any pharmacological or structural effect of the habit. We note that had chewers fared worse, behaviour and biology would have been indistinguishable as explanations, and that the null result renders the question less pressing but does not resolve it: the study cannot say whether a chewer who genuinely followed the behavioural advice would have done better than one who did not.

5. Mechanistic language

We agree. The previous version of the Discussion already stated that no cathinone assay, muscle-activity measurement or drug level was obtained and that response was not examined by drug class. We have strengthened the surrounding language so that the hypothetical status of these mechanisms is maintained throughout rather than acknowledged only once. The parsimonious reading is now introduced as "one that remains conjectural"; the clinical prediction that follows is now expressed in the conditional, with the explicit addition that the present study was not designed to test it and that nothing here establishes it. The comparison with the imaging literature now notes that the bimodal distribution observed here has no counterpart in it.

6. Typographical errors

The manuscript has been proofread in full. The specific corrections are: a sentence in the Participants section that lacked a main verb ("A total number of 156 patients screened…") has been rewritten; the exclusion criteria, where the removal of parenthetical dashes had inverted the sense of the list, so that it appeared to name sources from which the pain did arise, have been restored to their intended sense; the inconsistent spellings "stabilization" and "dichotomized" have been brought into line with the British forms used elsewhere; an inconsistent instance of "Fig. 2" has been corrected to "Fig 2"; and a sentence in the Clinical implications section in which the grammatical subject was obscured by an unpunctuated list has been revised.

Reviewer #3

We thank the reviewer for the assessment and for three points that usefully complement those raised by Reviewer 1.

1. Methodological limitations and power

We agree; this point has been addressed together with Reviewer 1's first point. Both the abstract and the Conclusion now state the power limitation explicitly and in terms of what the study cannot exclude rather than what it failed to find. The abstract closes with this statement, and the Conclusion raises it immediately after the primary result, before any interpretation is offered. We have also removed the phrase "no evidence emerged" from positions where it could be read as a positive finding of no effect, and replaced the Conclusion's opening claim with "no association was detected", which describes the observation rather than the underlying truth.

2. Blinding and examiner bias

We agree. The absence of blinding was previously described in the Methods but only briefly mentioned in the Discussion. The Discussion now includes a dedicated paragraph evaluating its potential impact. The paragraph makes three points: that calibration was strong for the physical signs but cannot correct a bias of expectation, and that the outcome in question was a judgement rather than a measurement; that the departmental expectation was that chewers fare worse, so any such bias would have widened the apparent gap rather than narrowed it, which makes a null finding harder to attribute to this source than a positive one would have been; and that the absence of blinding nonetheless remains a genuine weakness, and that a blinded assessor would be the most important improvement in any replication of this study.

3. Treatment adherence and sub-modalities

We agree and have addressed separately the two questions this point raises. On sub-modalities, Table 3 already reported every component by group, and the Results now state the inference explicitly: no component differed by exposure group, so neither the overall intensity of treatment nor the choice among its sub-modalities can account for the outcome comparison that follows. On compliance, the Results now state plainly that adherence is a separate question the dataset cannot answer and direct the reader to the Limitations, where it is discussed in detail in response to Reviewer 1's fourth point.

Summary of changes to the manuscript

1. The severity finding has been corrected from "greater pain severity" to a bimodal distribution with a conditional contrast among those in pain; rank-sum and cut-point analyses added to Methods, Results and Table 2.

2. Power framed as an inability to exclude a clinically meaningful difference, in the abstract, Results, Discussion and Conclusion.

3. “Exploratory” replaced by “hypothesis-generating” throughout, including Table 5 and the Fig 2 legend.

4. Methods now explain why the continuous NRS and millimetre values cannot be analysed.

5. Limitations restructured into four that bear on interpretation — power, severity measurement, symptom duration, adherence — followed by the remainder.

6. Symptom duration reframed as a limitation on the primary comparison.

7. Adherence foregrounded, with the soft-diet contradiction stated explicitly.

8. Mechanistic claims made conditional throughout the Discussion.

9. A dedicated Discussion paragraph weighing examiner non-blinding and the likely direction of its bias.

10. Results state that no treatment sub-modality differed between groups.

11. Reference list checked against PubMed for retractions; none found; list unchanged.

12. Full proofread, with the specific corrections listed above.

13. Data availability statement made precise: the analysis code reproduces every estimate derived from individual-level data, and the one descriptive figure that comes instead from the parent project as an aggregate, the distribution of chewing duration across bands, is identified as such. The description of the age variable has been corrected to the bands actually used.

14. The ethics statement has been repaired. A sentence covering consent for participants under 18 years had lost its grammatical structure during an earlier revision; it is now two sentences, with no change to what is stated.

We hope that these revisions satisfactorily address the reviewers’ concerns, and we thank them again for comments that have improved both the accuracy and the presentation of the paper.
