# Supplementary material for: Habitual qat chewing and short-term response to conservative management of temporomandibular disorders: A prospective cohort study in conflict-affected Yemen
Source: PLoS One. 2026 Nov 13;21(11):e0362744. doi: 10.1371/journal.pone.0362744 (PMC13766270; doi:10.1371/journal.pone.0362744)
Supplement: Supplementary file 3 [file pone.0362744.s001.docx]

**S1 Checklist. STROBE Statement — checklist of items that should be included in reports of cohort studies.**

*References are keyed by section heading to the clean revised manuscript (Manuscript.docx) rather than by page number, since pagination changes on typesetting. Where an item required substantive new content rather than a cross-reference, that content is described. This checklist was revised alongside the present round of revision and reflects the manuscript as resubmitted.*

| **Section / Topic** | **Item** | **Recommendation** | **How and where addressed in this revision** |
| --- | --- | --- | --- |
| **Title and abstract** | **1** | (a) Indicate the study's design with a commonly used term in the title or the abstract. (b) Provide in the abstract an informative and balanced summary of what was done and what was found. | Title names the design (“A Prospective Cohort Study”). The abstract is unstructured, as PLOS ONE requires, and states the design, setting, recruitment period, exposure definition, outcome, the principal effect estimates with 95% confidence intervals and the null primary result. [Title page; Abstract] |
| **Introduction — Background/rationale** | **2** | Explain the scientific background and rationale for the investigation being reported. | Introduction, first four paragraphs: TMD epidemiology and aetiological framework; the Yemeni conflict setting and its psychological burden; qat pharmacology and chewing behaviour; and the 2026 systematic review and meta-analysis [46], which establishes an association between chewing and TMD while containing no study of treatment response. [Introduction] |
| **Introduction — Objectives** | **3** | State specific objectives, including any prespecified hypotheses. | The final paragraph of the Introduction states the question and names the four covariates prespecified for adjustment: age, sex, self-reported emotional stress and self-reported tooth grinding. [Introduction, final paragraph] |
| **Methods — Study design** | **4** | Present key elements of study design early in the paper. | “Study design and setting” states a prospective observational cohort of consecutive patients receiving routine care, and states that no aspect of management was assigned by the investigators. [Methods — Study design and setting] |
| **Methods — Setting** | **5** | Describe the setting, locations, and relevant dates, including periods of recruitment, exposure, follow-up, and data collection. | Oral Medicine Department, Sana'a University and Al-Kuwait Teaching Hospital; recruitment 1 October 2018 to 31 October 2020; follow-up at 3 and 6 weeks and 3 months. [Methods — Study design and setting; Outcome] |
| **Methods — Participants** | **6** | (a) Give the eligibility criteria, and the sources and methods of selection of participants. Describe methods of follow-up. (b) For matched studies, give matching criteria and number of exposed and unexposed. | (a) Eligibility and exclusion criteria given in full; consecutive sampling; follow-up schedule stated. (b) Not a matched study; 54 exposed and 94 unexposed. [Methods — Participants] |
| **Methods — Variables** | **7** | Clearly define all outcomes, exposures, predictors, potential confounders, and effect modifiers. Give diagnostic criteria, if applicable. | Exposure defined as qat use in three or more sessions per week by structured interview, with duration recorded in three bands and the habitual chewing side; session duration and quantity chewed were not recorded. Outcome defined as the four-point global rating of change at three months, dichotomised as improved versus not improved. The four prespecified confounders and the RDC/TMD Axis I groups [45] are defined. Pain intensity and mandibular opening were transcribed into the dataset as ordered categories only, a constraint stated where the variables are defined. [Methods — Exposure and covariates; Outcome; Diagnostic criteria and clinical examination] |
| **Methods — Data sources/measurement** | **8** | For each variable of interest, give sources of data and details of methods of assessment. Describe comparability of assessment methods if there is more than one group. | Structured interview for exposure, sociodemographic variables and covariates; RDC/TMD Axis I examination by a single oral medicine specialist for signs; 11-point Numeric Rating Scale for pain, transcribed in bands; millimetre ruler between the central incisal edges, with the vertical incisal overlap added, for mandibular opening, transcribed as threshold categories. One examiner assessed both groups identically at both visits, and intra-examiner agreement is reported (kappa above 0.85 for all dichotomous items). [Methods — Diagnostic criteria and clinical examination; Exposure and covariates; Outcome] |
| **Methods — Bias** | **9** | Describe any efforts to address potential sources of bias. | Non-blinding of the single examiner to exposure status is stated explicitly where the examination is described, and the likely direction of the resulting bias, together with the reason it does not readily generate a null result, is argued in the Discussion. Confounding by indication is addressed by comparing every treatment component between groups (Table 3); confounding by measured covariates is addressed by the Firth penalised models. [Methods — Diagnostic criteria and clinical examination; Results — Treatment received; Discussion — Strengths and limitations] |
| **Methods — Study size** | **10** | Explain how the study size was arrived at. | No a priori sample size calculation was performed; the sample comprises all eligible patients presenting over a 25-month recruitment window constrained by the security situation. Post-hoc precision is reported in place of a retrospective power calculation for the observed effect: 80% power at a two-sided alpha of 0.05 for an absolute difference of 16.1 percentage points or larger, and power of 0.26 for the 6.6-point difference observed. [Methods — Sample size] |
| **Methods — Quantitative variables** | **11** | Explain how quantitative variables were handled in the analyses. If applicable, describe which groupings were chosen and why. | Age was analysed as an ordered five-level band and entered into the models as a single ordinal term, with a chi-square test for trend across the bands. Pain intensity is reported in the conventional bands (0, 1-3, 4-6, 7-10) and was analysed as an ordered variable by Wilcoxon rank-sum, the proportional-odds assumption being examined by estimating the odds ratio at each of the three cut-points. Duration of qat chewing was recorded only in three bands and could not be linked to the outcome, so it is reported descriptively and not modelled. No continuous variable was available for analysis. [Methods — Exposure and covariates; Statistical analysis] |
| **Methods — Statistical methods** | **12** | (a) Describe all statistical methods, including those used to control for confounding. (b) Describe any methods used to examine subgroups and interactions. (c) Explain how missing data were addressed. (d) If applicable, explain how loss to follow-up was addressed. (e) Describe any sensitivity analyses. | (a) Fisher’s exact test for binary comparisons and the chi-square test where the table exceeded 2 × 2; chi-square test for trend across the ordered age bands; absolute risk difference with a 95% confidence interval; risk ratio with a Katz logarithmic interval; Wilson intervals for proportions; Wilcoxon rank-sum across the ordered pain bands with cut-point odds ratios to test proportional odds; Firth penalised logistic regression with profile penalised-likelihood intervals and penalised likelihood-ratio tests for the adjusted estimates, with the adjustment set and the events-per-variable ratio stated for each model. Software and versions are named. (b) Sex-stratified estimates with a Mantel-Haenszel summary odds ratio; variance inflation factors reported. (c) No missing data arose in any analysed variable, so no imputation or complete-case rule was required. (d) Not applicable: a single fixed three-month follow-up with no loss. (e) Sensitivity analysis excluding the seven internally discordant records. [Methods — Statistical analysis] |
| **Results — Participants** | **13** | (a) Report numbers of individuals at each stage. (b) Give reasons for non-participation at each stage. (c) Consider use of a flow diagram. | (a) 156 screened, 8 not eligible, 148 enrolled and diagnosed, 148 followed and 148 analysed, with no loss to follow-up. (b) The number failing the eligibility criteria is reported; the dataset does not record which criterion each of the eight failed, so no breakdown by reason is given and none is implied. (c) Flow diagram provided as Fig 1. [Results — Participants and baseline characteristics; Fig 1] |
| **Results — Descriptive data** | **14** | (a) Give characteristics of study participants and information on exposures and potential confounders. (b) Indicate number of participants with missing data for each variable of interest. (c) Summarise follow-up time. | (a) Tables 1 and 2 give sociodemographic characteristics, presenting complaints, covariates and Axis I diagnoses by exposure group. (b) No missing data for any analysed variable. (c) Follow-up was fixed at three months and completed by all 148 participants. [Results — Participants and baseline characteristics; Tables 1-2] |
| **Results — Outcome data** | **15** | Report numbers of outcome events or summary measures over time. | Improvement occurred in 47 of 54 chewers and 88 of 94 non-chewers, giving 13 non-improvement events in total; the events-per-variable ratio implied for each model is stated. Reported in the text, in Table 4 and in Fig 2A. [Results — Short-term response; Table 4; Fig 2] |
| **Results — Main results** | **16** | (a) Give unadjusted estimates and, if applicable, confounder-adjusted estimates and their precision. State which confounders were adjusted for and why. (b) Report category boundaries when continuous variables were categorised. (c) If relevant, consider translating estimates of relative risk into absolute risk. | (a) Unadjusted risk difference, risk ratio, odds ratio and Fisher exact p-value; adjusted odds ratios from two Firth models, with the adjustment set stated and justified and the estimates labelled hypothesis-generating. (b) Age bands and Numeric Rating Scale band boundaries are given wherever the categories are reported. (c) The absolute risk difference of −6.6 percentage points (95% CI −16.8 to 3.7) is reported alongside the relative measures. [Results — Short-term response; Multivariable analysis (hypothesis-generating); Tables 4-5] |
| **Results — Other analyses** | **17** | Report other analyses done — e.g. analyses of subgroups and interactions, and sensitivity analyses. | Sex-stratified estimates and the Mantel-Haenszel odds ratio; variance inflation factors; the chi-square test for trend across age bands; the ordinal reanalysis of the baseline pain bands, comprising the rank-sum test and the three cut-point odds ratios, together with the analysis restricted to the 122 participants reporting any pain; and the sensitivity analysis excluding the seven discordant records. Each is identified as secondary or exploratory, and none is presented as confirmatory. [Results — Participants and baseline characteristics; Short-term response; Multivariable analysis (hypothesis-generating); Methods — Statistical analysis] |
| **Discussion — Key results** | **18** | Summarise key results with reference to study objectives. | The Discussion opens by summarising the three key results against the stated objective: the null primary comparison, age as the variable that accounts for the crude difference, and the bimodal rather than uniformly elevated distribution of baseline pain among chewers. [Discussion] |
| **Discussion — Limitations** | **19** | Discuss limitations of the study, taking into account sources of potential bias or imprecision. Discuss both direction and magnitude of any potential bias. | Limitations are discussed in a dedicated subsection and cover the limited power and what the confidence interval does not exclude; the single unvalidated global outcome and the absence of change scores, pain intensity and mandibular opening having been transcribed only as categories; the unblinded single examiner and the direction of that bias; unvalidated single-item stress and bruxism measures; coarse self-reported exposure without session duration or quantity, and duration bands that could not be linked to outcome; unmeasured confounders, among them symptom duration before presentation, tobacco smoking, shammah use and socioeconomic position beyond a three-level rating; unquantified adherence; the absence from the study database of dose, dosing frequency, treatment duration, splint specification and adverse-event data; the use of RDC/TMD rather than DC/TMD; and an events-per-variable ratio of 2.6 in the full model. [Discussion — Strengths and limitations] |
| **Discussion — Interpretation** | **20** | Give a cautious overall interpretation of results considering objectives, limitations, multiplicity of analyses, results from similar studies, and other relevant evidence. | Interpretation is framed associationally throughout, and the null result is read as absence of evidence rather than evidence of absence. The cytochrome P450 hypothesis is explicitly labelled as raised by the setting rather than supported by these data; the competing explanation of differential adherence is stated and weighed; and the findings are compared with the 2026 meta-analysis and with the single previous prospective study. [Discussion] |
| **Discussion — Generalisability** | **21** | Discuss the generalisability (external validity) of the study results. | Generalisability to other qat-prevalent settings is addressed, and the limits imposed by a single-centre, treatment-seeking, predominantly young and female sample are described where the cohort is characterised and again among the limitations. [Discussion — Clinical implications; Strengths and limitations; Results — Participants and baseline characteristics] |
| **Other information — Funding** | **22** | Give the source of funding and the role of the funders for the present study and, if applicable, for the original study on which the present article is based. | “The authors received no specific funding for this work.” The parent doctoral project received no external funding, and no funder had any role in study design, data collection, analysis, the decision to publish or preparation of the manuscript. [Funding] |

Reference: von Elm E, Altman DG, Egger M, Pocock SJ, Gøtzsche PC, Vandenbroucke JP; STROBE Initiative. The Strengthening the Reporting of Observational Studies in Epidemiology (STROBE) statement: guidelines for reporting observational studies. Lancet. 2007;370(9596):1453–1457.
