# Supplementary material for: Habitual qat chewing and short-term response to conservative management of temporomandibular disorders: A prospective cohort study in conflict-affected Yemen
Source: PLoS One. 2026 Nov 13;21(11):e0362744. doi: 10.1371/journal.pone.0362744 (PMC13766270; doi:10.1371/journal.pone.0362744)
Supplement: Supplementary file 4 [file pone.0362744.s003.docx]

Response to Editorial Ethical Concerns

Editorial Comment:

Concerns were raised regarding discrepancies in recruitment dates and lack of disclosure of prior submission.

Response:

We thank the Editorial Office for highlighting this important issue. We clarify that ethical approval for the study was obtained in September 2018, whereas patient recruitment began in October 2018. The earlier date reflects the approval by the Medical Ethics Committee, while recruitment was initiated only after completion of all preparatory steps. This distinction was not sufficiently clarified in the previous version and has now been explicitly stated in the Methods and Ethics sections of the manuscript.

We also acknowledge that the previous submission to PLOS ONE was not explicitly mentioned. We apologize for this oversight and confirm that the current submission is a revised version of the previously rejected manuscript, with full transparency provided in the cover letter and response document.

These clarifications do not affect the study data, analysis, or conclusions, but ensure full ethical and procedural transparency.
