# Supplementary material for: Habitual qat chewing and short-term response to conservative management of temporomandibular disorders: A prospective cohort study in conflict-affected Yemen
Source: PLoS One. 2026 Nov 13;21(11):e0362744. doi: 10.1371/journal.pone.0362744 (PMC13766270; doi:10.1371/journal.pone.0362744)
Supplement: Peer review history

# Author comment: Author response to Decision Letter 0

**DOI:** 10.1371/journal.pone.0362744.r001

8 Mar 2026

Attachment Submitted filename: Response To Reviewers 2026.docx

# Peer review: Decision Letter 0

**DOI:** 10.1371/journal.pone.0362744.r002

**Authors:** Eman Allam

4 Aug 2026

Dear Dr. Hajeb,

Thank you for submitting your manuscript to PLOS ONE. After careful consideration, we feel that it has merit but does not fully meet PLOS ONE’s publication criteria as it currently stands. Therefore, we invite you to submit a revised version of the manuscript that addresses the points raised during the review process.

Please submit your revised manuscript by Sep 18 2026 11:59PM. If you will need more time than this to complete your revisions, please reply to this message or contact the journal office at plosone@plos.org. When you're ready to submit your revision, log on to https://www.editorialmanager.com/pone/ and select the 'Submissions Needing Revision' folder to locate your manuscript file.

- A letter that responds to each point raised by the academic editor and reviewer(s). You should upload this letter as a separate file labeled 'Response to Reviewers'.
- A marked-up copy of your manuscript that highlights changes made to the original version. You should upload this as a separate file labeled 'Revised Manuscript with Track Changes'.
- An unmarked version of your revised paper without tracked changes. You should upload this as a separate file labeled 'Manuscript'.

If you would like to make changes to your financial disclosure, please include your updated statement in your cover letter. Guidelines for resubmitting your figure files are available below the reviewer comments at the end of this letter.

If applicable, we recommend that you deposit your laboratory protocols in protocols.io to enhance the reproducibility of your results. Protocols.io assigns your protocol its own identifier (DOI) so that it can be cited independently in the future. For instructions see: https://journals.plos.org/plosone/s/submission-guidelines#loc-laboratory-protocols. Additionally, PLOS ONE offers an option for publishing peer-reviewed Lab Protocol articles, which describe protocols hosted on protocols.io. Read more information on sharing protocols at https://plos.org/protocols?utm_medium=editorial-email&utm_source=authorletters&utm_campaign=protocols.

As the corresponding author, your ORCID iD is verified in the submission system and will appear in the published article. PLOS supports the use of ORCID, and we encourage all coauthors to register for an ORCID iD and use it as well. Please encourage your coauthors to verify their ORCID iD within the submission system before final acceptance, as unverified ORCID iDs will not appear in the published article. *Only*  the individual author can complete the verification step; PLOS staff *cannot*  verify ORCID iDs on behalf of authors.

We look forward to receiving your revised manuscript.

Kind regards,

Eman Allam, PhD

Academic Editor

PLOS One

Journal Requirements:

When submitting your revision, we need you to address these additional requirements.

1. Please ensure that your manuscript meets PLOS ONE's style requirements, including those for file naming. The PLOS ONE style templates can be found at

https://journals.plos.org/plosone/s/file?id=wjVg/PLOSOne_formatting_sample_main_body.pdf and

https://journals.plos.org/plosone/s/file?id=ba62/PLOSOne_formatting_sample_title_authors_affiliations.pdf

2. We note that you have indicated that there are restrictions to data sharing for this study. For studies involving human research participant data or other sensitive data, we encourage authors to share de-identified or anonymized data. However, when data cannot be publicly shared for ethical reasons, we allow authors to make their data sets available upon request. For information on unacceptable data access restrictions, please see http://journals.plos.org/plosone/s/data-availability#loc-unacceptable-data-access-restrictions.

Before we proceed with your manuscript, please address the following prompts:

a) If there are ethical or legal restrictions on sharing a de-identified data set, please explain them in detail (e.g., data contain potentially identifying or sensitive patient information, data are owned by a third-party organization, etc.) and who has imposed them (e.g., a Research Ethics Committee or Institutional Review Board, etc.). Please also provide contact information for a data access committee, ethics committee, or other institutional body to which data requests may be sent.

b) If there are no restrictions, please upload the minimal anonymized data set necessary to replicate your study findings to a stable, public repository and provide us with the relevant URLs, DOIs, or accession numbers. Please see http://www.bmj.com/content/340/bmj.c181.long for guidelines on how to de-identify and prepare clinical data for publication. For a list of recommended repositories, please see https://journals.plos.org/plosone/s/recommended-repositories. You also have the option of uploading the data as Supporting Information files, but we would recommend depositing data directly to a data repository if possible.

Please update your Data Availability statement in the submission form accordingly.

3. In the online submission form, you indicated that “De-identified individual-level data can be made available upon reasonable request to the corresponding author (Dr. Raji Mansoor Hajeb, [email address]).”

All PLOS journals now require all data underlying the findings described in their manuscript to be freely available to other researchers, either 1. In a public repository, 2. Within the manuscript itself, or 3. Uploaded as supplementary information.

This policy applies to all data except where public deposition would breach compliance with the protocol approved by your research ethics board. If your data cannot be made publicly available for ethical or legal reasons (e.g., public availability would compromise patient privacy), please explain your reasons on resubmission and your exemption request will be escalated for approval.

4. PLOS requires an ORCID iD for the corresponding author in Editorial Manager on papers submitted after December 6th, 2016. Please ensure that you have an ORCID iD and that it is validated in Editorial Manager. To do this, go to ‘Update my Information’ (in the upper left-hand corner of the main menu), and click on the Fetch/Validate link next to the ORCID field. This will take you to the ORCID site and allow you to create a new iD or authenticate a pre-existing iD in Editorial Manager.

5. Your ethics statement should only appear in the Methods section of your manuscript. If your ethics statement is written in any section besides the Methods, please move it to the Methods section and delete it from any other section. Please ensure that your ethics statement is included in your manuscript, as the ethics statement entered into the online submission form will not be published alongside your manuscript.

6. Please include captions for your Supporting Information files at the end of your manuscript, and update any in-text citations to match accordingly. Please see our Supporting Information guidelines for more information: http://journals.plos.org/plosone/s/supporting-information.

7. If the reviewer comments include a recommendation to cite specific previously published works, please review and evaluate these publications to determine whether they are relevant and should be cited. There is no requirement to cite these works unless the editor has indicated otherwise.

[Note: HTML markup is below. Please do not edit.]

Reviewers' comments:

Reviewer's Responses to Questions

**Comments to the Author**

1. Is the manuscript technically sound, and do the data support the conclusions?

Reviewer #1: Partly

Reviewer #2: Partly

**********

2. Has the statistical analysis been performed appropriately and rigorously?

Reviewer #1: Yes

Reviewer #2: No

**********

3. Have the authors made all data underlying the findings in their manuscript fully available?

The PLOS Data policy

Reviewer #1: No

Reviewer #2: No

**********

4. Is the manuscript presented in an intelligible fashion and written in standard English?

Reviewer #1: Yes

Reviewer #2: No

**********

Reviewer #1: Comments to the Author

This is an interesting prospective study examining the effect of qat chewing on conservative TMD treatment outcomes in Yemen. The topic is clinically relevant and the conflict-affected setting adds unique value. However, I have several concerns that need to be addressed before this work can be considered for publication.

Major Issues:

The study used RDC/TMD rather than DC/TMD for diagnosis. Since data collection started in 2018 — four years after DC/TMD became the standard — the authors should justify this choice or acknowledge it as a limitation.

The biggest concern is the lack of any quantified baseline severity measures. No VAS, NRS, or measured maximum mouth opening was recorded. Without knowing how severe symptoms were at baseline, it is difficult to meaningfully assess "improvement." Saying symptoms were present/absent (Table 4) does not establish comparable severity between groups.

The outcome measures (improved yes/no) are not operationally defined. What exactly counts as "substantial reduction" of pain or "observable functional improvement"? This makes the findings difficult to reproduce or interpret.

The examiner was apparently not blinded to qat chewing status, which introduces bias — especially for the so-called "objective" improvement endpoint. Also, inter-rater reliability is reported, but with a single examiner, intra-rater reliability is what matters.

The gender imbalance is striking (74% male in qat group vs. 35% in non-chewers). Sex differences in TMD are well-established, and simple adjustment in a regression model may not adequately handle a 40-percentage-point difference. Sex-stratified analyses would strengthen the paper.

The logistic regression has 5 predictors with only 32 failure events — this is borderline for overfitting. A post-hoc power discussion would be helpful.

Minor Issues:

The dose-response analysis (Figure 2) is based on only 54 subjects. The smooth curve is potentially misleading — showing the actual data distribution would be more transparent.

The discussion speculates about cathinone-CYP interactions reducing drug efficacy, but no pharmacokinetic data were collected. This should be clearly framed as hypothesis-generating.

Several typos need correction: "ong-term" → "Long-term" (Discussion), truncated funding sentence, double period in Table 4 title.

Reference numbering appears misaligned from ref 42 onward, and the STAB criteria reference seems to be missing from the list.

A STROBE checklist should be included as supplementary material.

The ethics approval document lists a different study title than the manuscript. This should be clarified, especially given that this is a resubmission of a previously rejected manuscript.

Overall: The research question is worthwhile and the prospective design is commendable, but the absence of validated quantitative outcome measures and baseline severity data is a fundamental limitation that weakens the conclusions considerably. I would recommend major revision with particular attention to points 1–6 above.

Reviewer #2: Thank you for the opportunity to review this manuscript. The research question is a good one and locally important, the prospective design suits it. Moreover, I think there are issues fixable with information you probably already hold, so I am recommending major revision rather than rejection. My comments follow the order of the manuscript.

Introduction

• The claim that no prospective study exists is contradicted by your own reference 23. Bakhadher et al. (2018) is listed as a prospective study of khat chewing and temporomandibular joint disorder. Please explain how your study differs.

• The stated aim mentions 'potential confounders' without naming them. Please list them, so the reader can check the regression model against what you set out to adjust for.

Methods

• Is this really an observational study? The treatment protocol reads as though the investigators assigned it (drugs 'tailored according to clinical presentation', splints 'provided to patients exhibiting parafunctional signs'), yet the study is described as observational with no registration. Please state who decided each element of management and whether this was routine departmental care or study-directed. If it was study-directed, the design is a prospective single-arm interventional study and the editorial office should advise on registration.

• The outcome definitions are the main problem. 'Substantial reduction', 'observable functional improvement' and 'pain-free maximum interincisal opening' are not defined anywhere. There is no threshold, no measurement in millimetres, and no rule for combining pain, joint sounds and limited opening into one yes/no verdict. As it stands another team could not classify a single patient. Please give the exact rule you applied at the follow-up visit, the opening threshold in millimetres, and the instrument used to measure it.

• The endpoint you analysed is not the one you specified. The Methods define two separate primary outcomes; the Results introduce a composite requiring both. Please state in the Methods whether the composite was pre-specified and when it was defined.

• One examiner assessed both the exposure and the outcome, unblinded. The examiner who recorded qat chewing status at baseline also judged improvement at three months, so blinding was impossible. Please state this explicitly, discuss the likely direction of bias, and consider relabelling the outcome 'examiner-assessed' rather than 'objective'.

• 'Inter-rater reliability (Kappa > 0.85)' does not fit a single examiner. This appears to be intra-examiner reliability. Please give the exact value with its 95% CI, how many patients were re-examined, the interval between examinations, and which variables it covers.

• The diagnostic criteria and the citation do not match. You state RDC/TMD Axis I but cite Schiffman et al. 2014, which is the DC/TMD. Recruitment began in 2018, four years after the DC/TMD appeared. Please state which instrument you used, cite it correctly, and briefly justify the choice. Please also report the distribution of Axis I diagnoses (myofascial pain, arthralgia, disc displacement) by group, since prognosis differs by subtype and an imbalance would confound the comparison.

• Treatment was not uniform, and we are not told who received what. If chewers were more or less likely to get a splint, or the diazepam-amitriptyline combination rather than naproxen, the association you report is confounded by treatment. Please add a table of n (%) receiving each component by group, with doses, frequency and duration, and specify the splint (material, arch, coverage, wear instructions, review schedule).

• Adherence is said to have been monitored but is never reported. Please report adherence to behavioural advice, medication and splint wear in each group.

• The exposure measure is coarse. A binary at three sessions per week tells us little. Please report session duration, amount chewed, and chewing side if recorded, and state whether anyone reduced or stopped qat use during the three months. Chewing side matters particularly, because unilateral mastication is central to the mechanism you argue for in the Discussion.

• Several likely confounders were not measured. Tobacco smoking and shammah use, socioeconomic status, and above all the duration of TMD symptoms before presentation, which is among the strongest prognostic factors for conservative therapy. Please name these in the Limitations.

• Please add participant flow. How many were screened, eligible, enrolled, completed the three-month visit, and were lost, in each group, with reasons. Over 25 months in a difficult setting, differential attrition is a real possibility and at present it cannot be judged. A STROBE flow diagram would cover this.

• The pharmacological regimen needs a safety paragraph. Please state the intended duration of diazepam and amitriptyline, how patients were monitored for dependence and sedation, and whether any adverse events occurred. Please also confirm the amitriptyline dose of 20 mg, since 10 mg and 25 mg are the usual starting doses.

Results

• Report the subjective and objective outcomes separately. Both were specified in the Methods and both are claimed in the Abstract, but Table 2 shows only the composite.

• The risk ratio does not match your own counts. Please recheck and state which method you used for the interval.

• The regression model is probably overfitted. Thirty-two failures across five predictors is about 6.4 events per variable, below the usual minimum of ten. Please report a fit statistic and the C-statistic, and consider a smaller model or Firth penalised estimation. Since you describe the analysis as exploratory in the Methods, please carry that wording into the Results and Conclusion too.

• Sex is almost collinear with the exposure. 74.1% of chewers are male versus 34.9% of non-chewers. Please report variance inflation factors and add either a sex-stratified analysis or a sensitivity analysis in men only.

• The dose-response analysis needs rethinking. First, state the n used for the Spearman correlation: duration exists only for chewers (n = 54), and if non-chewers were entered as zero the correlation just restates the group difference. Second, a rank correlation between a continuous exposure and a binary outcome is not the right test; please model duration as a continuous predictor and report the odds ratio per five years with a trend test. Third, give the numerators and denominators behind the 58% and 78% figures, and include the missing 10-20 year band.

• Some participants apparently had no pain at baseline. Table 4 implies 25.9% of chewers and 15.1% of non-chewers. How was 'improvement in pain' scored for them, and how did they contribute to the composite?

Discussion and Conclusion

• The Discussion is one paragraph running to roughly two pages. Please break it into principal findings, comparison with previous work, mechanisms, contextual considerations, clinical implications, and limitations.

• The language is causal where the design is not. 'Appears to reduce the short-term effectiveness' and 'support a combined neuromuscular and pharmacokinetic explanation' go beyond what an observational cohort can show, particularly as no neuromuscular or pharmacokinetic variable was measured. Please reframe in associational terms and present the CYP interaction as a hypothesis your findings raise rather than one they support.

• The most obvious competing explanation is not addressed. Every participant was counselled to adopt a soft diet and avoid parafunctional loading. Continuing to chew qat daily is itself a departure from that advice, so the poorer outcome in chewers may reflect differential adherence to the behavioural component rather than any qat-specific biological effect. This needs to be raised and argued against directly, ideally with the adherence data requested above.

• The Limitations are honest but incomplete. Please add the unblinded single assessor, the absence of participant flow data, the unmeasured confounders listed above, and the low events-per-variable ratio in the model.

• COVID-19 overlapped the end of recruitment. Recruitment ran to 31 October 2020. Please say whether it was interrupted and whether follow-up completeness was affected.

References and language

• The citation numbering is out of step with the list. The Ethiopian undernutrition meta-analysis is cited as [43] but appears as reference 42; 'STAB criteria [44, 45]' cites a reference 45 that does not exist. The offset suggests the problem may run further, so please check the whole text-to-list mapping.

• One reference looks misplaced. Reference 36 (Abdulkader, 'The forgotten war') sits inside the cluster [31-36] supporting psychological factors in TMD, where it does not seem to belong. please recheck.

• Typographical recheck needed for whole manuscript.

• 'Qat' and 'khat' are used interchangeably. Please note the equivalence once, then keep to one spelling.

Ethics, data availability and reporting

• The data availability statement contradicts the manuscript. The submission system says the data are available 'upon reasonable request', which PLOS does not accept, while the Declarations say anonymised data are provided as S1 Data and an S1_data.xlsx is attached. If S1 Data holds the de-identified individual-level dataset behind every analysis, please update the submission statement to say so. If it holds only aggregate data, the individual-level data need to be deposited or supplied as Supporting Information.

• The two ethics statements differ. The submission form names only Sana'a University, with no approval number; the manuscript gives Approval No. 166 of 15 September 2018 and also names the Research Committee at National Ribat University. Please make the two identical, with every approving body, number and date.

• Please supply the STROBE checklist. It is required for observational studies, and several of the gaps above map directly onto its items.

I hope these comments are read as constructive. The clinical question deserves an answer and you are well placed to give one; what the manuscript needs is enough methodological detail for a reader to judge the finding, and conclusions pitched at the level the design supports. I would be glad to look at a revised version.

**********

what does this mean?). If published, this will include your full peer review and any attached files.

If you choose “no”, your identity will remain anonymous but your review may still be made public.

**Do you want your identity to be public for this peer review?**  For information about this choice, including consent withdrawal, please see our Privacy Policy

Reviewer #1: **Yes:**  Mohammed Elsaid

Reviewer #2: No

**********

[NOTE: If reviewer comments were submitted as an attachment file, they will be attached to this email and accessible via the submission site. Please log into your account, locate the manuscript record, and check for the action link "View Attachments". If this link does not appear, there are no attachment files.]

To ensure your figures meet our technical requirements, please review our figure guidelines: https://journals.plos.org/plosone/s/figures

You may also use PLOS’s free figure tool, NAAS, to help you prepare publication quality figures: https://journals.plos.org/plosone/s/figures#loc-tools-for-figure-preparation.

NAAS will assess whether your figures meet our technical requirements by comparing each figure against our figure specifications.

# Author comment: Author response to Decision Letter 1

**DOI:** 10.1371/journal.pone.0362744.r003

8 Sep 2026

Thank you for the opportunity to revise and resubmit our manuscript. We have carefully addressed all comments raised by the reviewers and the editor. A detailed point-by-point Response to Reviewers document has been uploaded with the revised submission. We have also revised the manuscript accordingly. We appreciate the reviewers’ and editor’s valuable comments and suggestions.

Attachment Submitted filename: Response to Reviewers.pdf

# Peer review: Decision Letter 1

**DOI:** 10.1371/journal.pone.0362744.r004

**Authors:** Eman Allam

9 Oct 2026

Dear Dr. Hajeb,

Thank you for submitting your manuscript to PLOS One. After careful consideration, we feel that it has merit but does not fully meet PLOS One’s publication criteria as it currently stands. Therefore, we invite you to submit a revised version of the manuscript that addresses the points raised during the review process.

Please submit your revised manuscript by Nov 23 2026 11:59PM. If you will need more time than this to complete your revisions, please reply to this message or contact the journal office at plosone@plos.org. When you're ready to submit your revision, log on to https://www.editorialmanager.com/pone/ and select the 'Submissions Needing Revision' folder to locate your manuscript file.

- A letter that responds to each point raised by the academic editor and reviewer(s). You should upload this letter as a separate file labeled 'Response to Reviewers'.
- A marked-up copy of your manuscript that highlights changes made to the original version. You should upload this as a separate file labeled 'Revised Manuscript with Track Changes'.
- An unmarked version of your revised paper without tracked changes. You should upload this as a separate file labeled 'Manuscript'.

If you would like to make changes to your financial disclosure, please include your updated statement in your cover letter. Guidelines for resubmitting your figure files are available below the reviewer comments at the end of this letter.

If applicable, we recommend that you deposit your laboratory protocols in protocols.io to enhance the reproducibility of your results. Protocols.io assigns your protocol its own identifier (DOI) so that it can be cited independently in the future. For instructions see: https://journals.plos.org/plosone/s/submission-guidelines#loc-laboratory-protocols. Additionally, PLOS One offers an option for publishing peer-reviewed Lab Protocol articles, which describe protocols hosted on protocols.io. Read more information on sharing protocols at https://plos.org/protocols?utm_medium=editorial-email&utm_source=authorletters&utm_campaign=protocols.

As the corresponding author, your ORCID iD is verified in the submission system and will appear in the published article. PLOS supports the use of ORCID, and we encourage all coauthors to register for an ORCID iD and use it as well. Please encourage your coauthors to verify their ORCID iD within the submission system before final acceptance, as unverified ORCID iDs will not appear in the published article. *Only*  the individual author can complete the verification step; PLOS staff *cannot*  verify ORCID iDs on behalf of authors.

We look forward to receiving your revised manuscript.

Kind regards,

Eman Allam, PhD

Academic Editor

PLOS One

**Journal Requirements:**

1. If the reviewer comments include a recommendation to cite specific previously published works, please review and evaluate these publications to determine whether they are relevant and should be cited. There is no requirement to cite these works unless the editor has indicated otherwise.

2. Please review your reference list to ensure that it is complete and correct. If you have cited papers that have been retracted, please include the rationale for doing so in the manuscript text, or remove these references and replace them with relevant current references. Any changes to the reference list should be mentioned in the rebuttal letter that accompanies your revised manuscript. If you need to cite a retracted article, indicate the article’s retracted status in the References list and also include a citation and full reference for the retraction notice.

[Note: HTML markup is below. Please do not edit.]

Reviewers' comments:

Reviewer's Responses to Questions

**Comments to the Author**

Reviewer #1: (No Response)

Reviewer #3: (No Response)

**********

2. Is the manuscript technically sound, and do the data support the conclusions?

Reviewer #1: Yes

Reviewer #3: Yes

**********

3. Has the statistical analysis been performed appropriately and rigorously?

Reviewer #1: Yes

Reviewer #3: Yes

**********

4. Have the authors made all data underlying the findings in their manuscript fully available?

The PLOS Data policy

Reviewer #1: Yes

Reviewer #3: Yes

**********

5. Is the manuscript presented in an intelligible fashion and written in standard English?

Reviewer #1: Yes

Reviewer #3: Yes

**********

Reviewer #1: The authors have substantially strengthened this manuscript. I particularly commend their transparency in identifying and correcting significant errors in the original submission and in re-running the entire analysis, which appropriately changed the central conclusion to a null finding. Provision of the de-identified dataset, analysis code, and STROBE checklist now allows full reproducibility, and I was able to follow the corrected analytic logic. The revised framing — that habitual qat chewing was not associated with poorer three-month response, with the crude difference explained by age — is well supported and appropriately cautious.

A few points remain:

Power and interpretation. The study can detect only differences of ≥16 percentage points, whereas the observed difference was 6.6 points. Please ensure every statement of "no association" is explicitly framed as "unable to exclude a clinically meaningful difference," including in the abstract conclusion. All multivariable models rest on only 13 non-improvement events and should be labeled hypothesis-generating throughout, not only in the limitations.

Baseline severity data. The manuscript notes that baseline pain (NRS) and mandibular opening were collected but not entered into the analysis dataset. This is the most consequential remaining gap, since the more novel finding — that chewers present with greater pain severity — rests on a categorical severity variable rather than the continuous measures that were reportedly obtained. Please clarify why the continuous baseline (and any follow-up) measures cannot be analyzed, or incorporate them.

Unmeasured confounders. The added limitations (tobacco/shammah use, socioeconomic detail, and especially symptom duration before presentation) are appropriate. Given that symptom duration is among the strongest prognostic factors for conservative therapy and was not captured, please state this explicitly as a limitation on the primary comparison, not only in passing.

Adherence. Since chewing itself violates the soft-diet advice central to the intervention, the absence of an analyzable adherence variable should be foregrounded as a limitation directly relevant to interpreting the null result.

Mechanistic language. The cathinone/CYP pharmacokinetic and neuromuscular mechanisms remain untested here (no drug levels; response did not differ by drug class). Please keep this clearly hypothetical wherever it appears in the Discussion.

Proofread for residual typographical errors carried over from the prior version.

These are refinements rather than fundamental concerns. With attention to the above, the manuscript is suitable for publication.

Reviewer #3: This is an interesting and well-conducted prospective cohort study investigating the short-term outcomes of conservative treatment for temporomandibular disorders (TMD) among habitual qat chewers and non-chewers. The manuscript addresses a relevant gap in the literature by evaluating actual treatment response rather than just prevalence. The authors are to be commended for achieving a 100% follow-up rate at 3 months, and the application of Firth-penalized logistic regression appropriately addresses the challenges of small event numbers.

To further strengthen the manuscript, I suggest the following minor considerations:

1. Methodological Limitations & Power: As openly acknowledged, the study is underpowered to detect minor differences between groups. This should be emphasized clearly in the abstract and conclusion to avoid overinterpretation of the negative finding regarding qat chewing.

2. Blinding and Examiner Bias: The study relies on a single examiner who was not blinded to the patients' qat-chewing habits during follow-up assessments. While baseline calibration was strong (Cohen's kappa > 0.85), this limitation should be explicitly weighed in the discussion.

3. Treatment Adherence: A brief note on whether treatment compliance or specific sub-modalities differed between groups would add helpful clinical context.

Overall, this is a valuable contribution to oral medicine.

**********

what does this mean?). If published, this will include your full peer review and any attached files.

If you choose “no”, your identity will remain anonymous but your review may still be made public.

**Do you want your identity to be public for this peer review?**  For information about this choice, including consent withdrawal, please see our Privacy Policy

Reviewer #1: **Yes:**  Mohammed Elsaid

Reviewer #3: **Yes:**  Pål Barkvoll

**********

[NOTE: If reviewer comments were submitted as an attachment file, they will be attached to this email and accessible via the submission site. Please log into your account, locate the manuscript record, and check for the action link "View Attachments". If this link does not appear, there are no attachment files.]

To ensure your figures meet our technical requirements, please review our figure guidelines: https://journals.plos.org/plosone/s/figures

You may also use PLOS’s free figure tool, NAAS, to help you prepare publication quality figures: https://journals.plos.org/plosone/s/figures#loc-tools-for-figure-preparation.

NAAS will assess whether your figures meet our technical requirements by comparing each figure against our figure specifications.

# Author comment: Author response to Decision Letter 2

**DOI:** 10.1371/journal.pone.0362744.r005

19 Oct 2026

We thank the Editor and both reviewers for their careful evaluation of our revised manuscript and for their constructive comments. We have addressed the reviewers’ concerns and revised the manuscript accordingly.

In particular, we corrected the interpretation of baseline pain severity, clarified the study’s statistical power and limitations, explained why the original continuous measurements were unavailable for analysis, and strengthened the discussion of potential confounding, treatment adherence, examiner blinding, and the hypothetical nature of the proposed mechanisms. We also clarified the interpretation of the multivariable analyses, revised the relevant tables and figures, corrected typographical and grammatical errors, and improved the data availability and ethics statements.

A detailed, point-by-point response to all reviewer comments and a summary of the corresponding manuscript revisions are provided in the attached Response to Reviewers document.

We sincerely appreciate the Editor’s and reviewers’ feedback, which has helped us improve the accuracy, transparency, and clarity of the manuscript.

Attachment Submitted filename: 1792176401691_Response to Reviewers V2.pdf

# Peer review: Decision Letter 2

**DOI:** 10.1371/journal.pone.0362744.r006

**Authors:** Eman Allam

30 Oct 2026

Habitual qat chewing and short-term response to conservative management of temporomandibular disorders: a prospective cohort study in conflict-affected Yemen

PONE-D-26-07488R2

Dear Dr. Hajeb,

We’re pleased to inform you that your manuscript has been judged scientifically suitable for publication and will be formally accepted for publication once it meets all outstanding technical requirements.

Within one week, you’ll receive an e-mail detailing the required amendments. When these have been addressed, you’ll receive a formal acceptance letter and your manuscript will be scheduled for publication.

An invoice will be generated when your article is formally accepted. Please note, if your institution has a publishing partnership with PLOS and your article meets the relevant criteria, all or part of your publication costs will be covered. Please make sure your user information is up-to-date by logging into Editorial Manager at Editorial Manager® and clicking the ‘Update My Information' link at the top of the page. For questions related to billing, please contact billing support.

If your institution or institutions have a press office, please notify them about your upcoming paper to help maximize its impact. If they’ll be preparing press materials, please inform our press team as soon as possible -- no later than 48 hours after receiving the formal acceptance. Your manuscript will remain under strict press embargo until 2 pm Eastern Time on the date of publication. For more information, please contact onepress@plos.org.

Kind regards,

Eman Allam, PhD

Academic Editor

PLOS One

Additional Editor Comments (optional):

Reviewers' comments:

Reviewer's Responses to Questions

**Comments to the Author**

Reviewer #1: All comments have been addressed

Reviewer #3: All comments have been addressed

**********

2. Is the manuscript technically sound, and do the data support the conclusions?

Reviewer #1: Yes

Reviewer #3: Yes

**********

3. Has the statistical analysis been performed appropriately and rigorously?

Reviewer #1: Yes

Reviewer #3: Yes

**********

4. Have the authors made all data underlying the findings in their manuscript fully available?

The PLOS Data policy

Reviewer #1: Yes

Reviewer #3: Yes

**********

5. Is the manuscript presented in an intelligible fashion and written in standard English?

Reviewer #1: Yes

Reviewer #3: Yes

**********

Reviewer #1: (No Response)

Reviewer #3: The authors are commended for their comprehensive and constructive revisions in this second revision (R2). They have thoroughly addressed previous concerns and significantly improved the clarity, methodological transparency, and overall quality of the manuscript.

Key improvements in this version include:

Statistical Power and Conclusions: The manuscript now appropriately frames the findings as hypothesis-generating, explicitly acknowledging the limited statistical power due to the small number of non-improvement events. This prevents overinterpretation of the negative findings regarding qat chewing.

Baseline Data and Measurement Methodology: The authors have clearly explained the handling of baseline measurements and the shift to ordinal/categorized data within the electronic records, enhancing methodological transparency.

Confounding Factors and Limitations: Important contextual factors, including baseline pain severity, patient age, high background emotional stress, lack of outcome blinding, and unquantified treatment adherence, are now candidly discussed within the study's limitations.

Presentation and Transparency: The point-by-point response is thorough, tables and figures have been properly updated, and the underlying dataset and analysis scripts remain fully accessible.

**********

what does this mean?). If published, this will include your full peer review and any attached files.

If you choose “no”, your identity will remain anonymous but your review may still be made public.

**Do you want your identity to be public for this peer review?**  For information about this choice, including consent withdrawal, please see our Privacy Policy

Reviewer #1: No

Reviewer #3: No

**********

# Editor report: Acceptance letter

**DOI:** 10.1371/journal.pone.0362744.r007

**Authors:** Eman Allam

PONE-D-26-07488R2

PLOS One

Dear Dr. Hajeb,

I'm pleased to inform you that your manuscript has been deemed suitable for publication in PLOS One. Congratulations! Your manuscript is now being handed over to our production team.

At this stage, our production department will prepare your paper for publication. This includes ensuring the following:

* All references, tables, and figures are properly cited

* All relevant supporting information is included in the manuscript submission,

* There are no issues that prevent the paper from being properly typeset

You will receive further instructions from the production team, including instructions on how to review your proof when it is ready. Please keep in mind that we are working through a large volume of accepted articles, so please give us a few days to review your paper and let you know the next and final steps.

Lastly, if your institution or institutions have a press office, please let them know about your upcoming paper now to help maximize its impact. If they'll be preparing press materials, please inform our press team within the next 48 hours. Your manuscript will remain under strict press embargo until 2 pm Eastern Time on the date of publication. For more information, please contact onepress@plos.org.

You will receive an invoice from PLOS for your publication fee after your manuscript has reached the completed accept phase. If you receive an email requesting payment before acceptance or for any other service, this may be a phishing scheme. Learn how to identify phishing emails and protect your accounts at https://explore.plos.org/phishing.

If we can help with anything else, please email us at customercare@plos.org.

Thank you for submitting your work to PLOS One and supporting open access.

Kind regards,

PLOS One Editorial Office Staff

on behalf of

Dr. Eman Allam

Academic Editor

PLOS One
